# Success of Escherichia coli O25b:H4 Sequence Type 131 Clade C Associated with a Decrease in Virulence

**DOI:** 10.1128/IAI.00576-20

**Published:** 2020-11-16

**Authors:** Marion Duprilot, Alexandra Baron, François Blanquart, Sara Dion, Cassandra Pouget, Philippe Lettéron, Saskia-Camille Flament-Simon, Olivier Clermont, Erick Denamur, Marie-Hélène Nicolas-Chanoine

**Affiliations:** a Université de Paris, INSERM, IAME, Paris, France; b AP-HP, Laboratoire de Microbiologie, Hôpital Beaujon, Clichy, France; c Centre for Interdisciplinary Research in Biology (CIRB), Collège de France, CNRS, INSERM, PSL Research University, Paris, France; d VBMI, INSERM U1047, Université de Montpellier, Nîmes, France; e Université de Paris, UMR 1149, INSERM-ERL, CNRS 8252, Paris, France; f Laboratorio de Referencia de Escherichia coli (LREC), Departamento de Microbioloxía e Parasitoloxía, Facultade de Veterinaria, Universidade de Santiago de Compostela (USC), Lugo, Spain; g AP-HP, Laboratoire de Génétique Moléculaire, Hôpital Bichat, Paris, France; University of California, Davis

**Keywords:** *Escherichia coli*, ST131, fimbriae, *ibeART* operon, *in vivo* virulence, niche adaptation

## Abstract

Escherichia coli O25b:H4 sequence type 131 (ST131), which is resistant to fluoroquinolones and which is a producer of CTX-M-15, is globally one of the major extraintestinal pathogenic E. coli (ExPEC) lineages. Phylogenetic analyses showed that multidrug-resistant ST131 strains belong to clade C, which recently emerged from clade B by stepwise evolution. It has been hypothesized that features other than multidrug resistance could contribute to this dissemination since other major global ExPEC lineages (ST73 and ST95) are mostly antibiotic susceptible. To test this hypothesis, we compared early biofilm production, presence of ExPEC virulence factors (VFs), and *in vivo* virulence in a mouse sepsis model in 19 and 20 epidemiologically relevant strains of clades B and C, respectively. Clade B strains were significantly earlier biofilm producers (*P* < 0.001), carriers of more VFs (*P* = 4e−07), and faster killers of mice (*P* = 2e−10) than clade C strains. Gene inactivation experiments showed that the *H*30-*fimB* and *ibeART* genes were associated with *in vivo* virulence. Competition assays in sepsis, gut colonization, and urinary tract infection models between the most anciently diverged strain (B1 subclade), one C1 subclade strain, and a B4 subclade recombining strain harboring some clade C-specific genetic events showed that the B1 strain always outcompeted the C1 strain, whereas the B4 strain outcompeted the C1 strain, depending on the mouse niches. All these findings strongly suggest that clade C evolution includes a progressive loss of virulence involving multiple genes, possibly enhancing overall strain fitness by avoiding severe infections, even if it comes at the cost of a lower colonization ability.

## Abstract (precis)

Escherichia coli O25b:H4 sequence type 131 (ST131), which is resistant to fluoroquinolones and which is a producer of CTX-M-15, is globally one of the major extraintestinal pathogenic E. coli (ExPEC) lineages. Phylogenetic analyses showed that multidrug-resistant ST131 strains belong to clade C, which recently emerged from clade B by stepwise evolution. It has been hypothesized that features other than multidrug resistance could contribute to this dissemination since other major global ExPEC lineages (ST73 and ST95) are mostly antibiotic susceptible.

## INTRODUCTION

In the last 2 decades, multidrug-resistant (MDR) Escherichia coli O25b:H4 sequence type 131 (ST131) has emerged worldwide among human extraintestinal pathogenic E. coli (ExPEC) strains (1, 2). This E. coli lineage is composed of two clades, called clades B and C, defined on the basis of the *fimH* alleles *H*22 and *H*30 (3, 4). A time-calibrated phylogeny showed that clade C emerged from clade B and that sequential mutational events shaped the evolution of O25b:H4 ST131 from the 1950s, resulting in the diversification of clade B into different subclades (subclades B1 to B5 and then the intermediate subclade, B0) and the appearance of clade C (5). The diversification of clade C into different subclades was characterized by the acquisition of the *fimH30* allele in about 1980, resulting in the C0 subclade, followed by the acquisition of the *gyrA1AB* and *parC1aAB* alleles, encoding fluoroquinolone resistance, in about 1987, resulting in the C1 and C2 subclades (5). All C2 subclade strains and a fraction of the C1 subclade strains forming the C1-M27 cluster (6) are additionally resistant to extended-spectrum cephalosporins (ESC) due to the production of extended-spectrum β-lactamases (ESBL) of the CTX-M type, CTX-M-15 and CTX-M-27, respectively.

Existing epidemiological studies have examined the prevalence of ST131 among E. coli isolates that are resistant to fluoroquinolones and/or that are producers of ESBL (7). Few studies have assessed the relative frequencies of ST131 clade B and clade C (8–11), and none of them have assessed the relative frequencies of the B and C subclades.

The evolutionary success of MDR ST131 clade C is still largely unexplained. Multidrug resistance may explain this success, as resistance to fluoroquinolones and ESC was shown to not impact the growth fitness of clade C strains (12, 13). However, E. coli lineages susceptible to antibiotics, such as ST73 and ST95, are as successful as ST131 clade C (in terms of their frequency in bacteremia and urinary tract infections), suggesting that factors other than antibiotic resistance can participate in the success of a lineage (9). Thus, by combining population genomics and modeling approaches, McNally et al. (14) showed that, compared to other dominant E. coli lineages and also to ST131 clade B, MDR ST131 clade C has accumulated a significantly elevated allelic diversity particularly enriched for genes involved in anaerobic metabolisms and other loci encoding factors contributing to human colonization by ExPEC. Given the low frequency of occurrence of this allelic diversity, the authors suggested adaptation to separate ecological niches among ST131 clades. For their part, Billard-Pomares et al. (15) showed that since 2000 the arginine deiminase operon has been increasing in prevalence in isolates producing ESBL, especially CTX-M, largely due to its presence in ST131 clade C. This operon was deleterious for the ESBL-producing strain tested in a mouse gut colonization model but contributed to its virulence in a mouse model of urinary tract infection (15). Therefore, this suggests the potential for ST131 clade C to adapt to different human niches.

In the present study, we chose to mostly perform phenotypic comparisons between clade B and clade C strains to get further insights into factors that have favored clade C dissemination. To this end, we established a collection of 39 E. coli O25b:H4 ST131 strains composed of strains representing the O25b:H4 ST131 clade and subclade diversity. The characterization of the 39 strains with regard to early biofilm formation and the capability to kill mice in a sepsis model, as well as competitions between selected clade B and clade C strains in various mouse models, allowed us to highlight the difference in the virulence potential of clade B and clade C strains.

## RESULTS

### Epidemiology of the O25b:H4 ST131 B and C subclades in extraintestinal infections over time.

**FIG 1:**
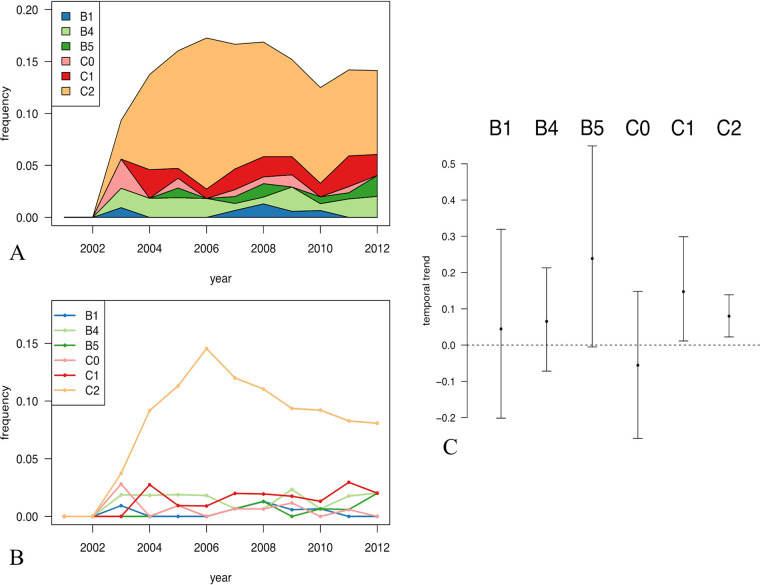
Dynamics of subclades in 197 O25b:H4 ST131 Escherichia coli strains isolated from cases of bacteremia over a 11-year sampling period (data are from Kallonen et al. [9]). (A) Cumulative frequencies of O25b ST131 subclades as a function of time, from the most ancestral B1 subclade at the bottom to the derived C2 subclade on the top. (B) Frequency of ST131 subclades as a function of time. Subclades are represented by colors. (C) Inferred linear trends of ST131 subclade frequencies (among all E. coli strains [*n* = 1,509]) as a function of time from 2001 to 2012. Among the clade B strains, only the subclade B5 strains exhibited a notable increase (logistic regression for subclade B5 strain presence as a function of time, 0.24 per year [95% CI, −0.005 to 0.55 per year]). Both the C1 and C2 subclade strains significantly increased (0.15 per year [95% CI, 0.01 to 0.30 per year] for C1, 0.08 per year [95% CI, 0.02 to 0.14 per year] for C2).

Recently, Kallonen et al. depicted 197 O25b:H4 ST131 isolates (161 belonging to clade C and 36 belonging to clade B) among 1,509 bacteremia E. coli isolates systematically collected between 2001 and 2012 in England (9). To have an overview of the epidemiology of the O25b:H4 ST131 B and C subclades, we used the whole-genome sequence of the English O25b:H4 ST131 isolates and reference strains of the different O25b:H4 ST131 B and C subclades (5) to build a phylogenetic tree from nonrecombinant single nucleotide polymorphisms (SNPs) of core genomes and clarify to which B and C subclades the 197 English O25b:H4 ST131 isolates belonged (see Fig. S1 in the supplemental material). We then assessed their relative frequencies (Fig. 1A and B) and investigated the trends in their frequency over the period from 2001 to 2012 (Fig. 1C). Among clade B strains, only the B5 subclade strains exhibited a notable increase (logistic regression for B5 strain presence as a function of time, 0.24 per year [95% confidence interval {CI}, −0.005 to 0.5 per year]). Both the C1 and C2 subclade strains significantly increased (0.15 per year [95% CI, 0.01 to 0.30 per year] for C1, 0.08 per year [95% CI, 0.02 to 0.14 per year] for C2).

### Molecular characteristics of the 39 studied O25b:H4 ST131 strains.

As illustrated in Fig. 1 and by studies that had used the *fimH* typing method to characterize O25b:H4 ST131 collections (16, 17), clade B/*fimH22* strains are much less frequent than clade C/*fimH30* strains. Therefore, to establish an O25b ST131 collection comprising as many clade B strains as clade C strains, we obtained *fimH22* strains either previously published or belonging to the coauthors’ personal collections (Table S1). Thus, we were able to get 18 *fimH22* strains, to which were added 21 *fimH30* strains, which generally came from the same collections as the *fimH22* strains (Table S1). The 39 strains were human strains isolated in Spain and France between 1993 and 2012 mostly from the blood of adult patients and the feces of healthy adults and children (Table S1). Through the phylogenetic tree constructed from nonrecombinant SNPs of core genome genes (Fig. S1), we found that the 39 strains were composed of 19 clade B strains distributed into the different B subclades, except for the B0 and B2 subclades (two subclades also absent among the 36 English O25b:H4 ST131 clade B isolates), and 20 clade C strains distributed into the three C subclades (subclades C0, C1, and C2). Among the 19 clade B strains, 17 displayed the *fimH22* allele; 1 displayed a *fimH22*-like allele; and 1, B4 subclade strain CES131C, displayed the *fimH30* allele that had been described to be specific to clade C strains (Table 1). For this reason, the CES131C strain is called “Recombinant” in the rest of this report.

**TABLE 1:** Genotypic and phenotypic characteristics of the 39 O25b:H4 ST131 Escherichia coli strains^*a*^

Strain	*fimH* operon type	Subclade	Virotype	β-Lactamase		Allele		Gene(s) encoding resistance to:		Plasmid Inc system	pMLST	Col-like plasmid	VF score	Biofilm phenotype
				Non-ESBL	ESBL	*gyrA*	*parC*	Aminoglycosides	Other antibiotics					
B11J12	*H*22	B1	D3	TEM-1B		*gyrA1A*	*parC1*	*aac(3)-IVa*, *aph(4)-Ia*		F1C, FIB	F89:A−:B62	156	16	−+
S250	*H*22	B1	D3			*gyrA1a*	*parC1*			F1C, FIB	F89:A−:B62		14	++
CES106C2	*H*22	B3	D4		CTX-M-1	*gyrA1a*	*parC2*		*sul2*, *tet*(A)	F1C, FIB, I1	F18:A−:B1, I1−ST3	pVC	16	++
H2381	*H*22	B3	D4	TEM-1C		*gyrA1A*	*parC2*		*tet*(A)	FII, FIB, X1	F2:A−:B1	MG828	18	++
H3345	*H*22	B3	D4	TEM-1C	SHV-12	*gyrA1A*	*parC2*			FII, FIB, FIC	F18:A−:B1	pVC	19	−+
H1659	*H*22	B4	D3			*gyrA1a*	*parC1*			FII, FIB	F29:A−:B10	156	16	−+
B7J19	*H*22	B4	D3			*gyrA1a*	*parC1*			FII, FIB	F29:A−:B10	156	16	−
B12C27	*H*22	B4	D3			*gyrA1a*	*parC1*			FII, FIB	F29:A−:B10	156, MG828	16	−
B5E11	*H*22	B4	D3			*gyrA1a*	*parC1*			FII, FIB	F29:A−:B10	156	16	−
011-005	*H*22	B4	D3			*gyrA1a*	*parC1*			FII, FIB	F29:A−:B10	156	15	−+
001-001	*H*22-like	B4	ND			*gyrA1a*	*parC1*			FII, FIB	F29:A−:B10	156	15	−+
CES131C	*H*30	B4	ND	TEM-1B		*gyrA1a*	*parC1*	*aac(3)-IId*, *aadA2*	*mph*(A), *sul1*, *tet*(A), *dfrA12*	FII, FIB	F2:A−:B10	BS512	15	−+
H219B	*H*22	B5	D1			*gyrA1a*	*parC1*			FII, FIB	F24:A−:B1		19	++
H1447	*H*22	B5	D1	TEM-1B		*gyrA1A*	*parC1*	*strA*, *strB*, *aadA5*	*mph*(B), *sul12*, *tet*(A), *dfrA1*	F1C, FB1, Q1	F18:A−:B8		17	−+
H1698	*H*22	B5	D2	TEM-1C		*gyrA1a*	*parC1*		*tet*(A)	FII, FIB, X1	F2:A−:B1		22	−+
196	*H*22	B5	D1	TEM-1B		*gyrA1a*	*parC1*	*aadA1*	*sul1*, *tet*(A), *dfrA1*	FII, FIB	F24:A−:B6		16	++
208	*H*22	B5	D1			*gyrA1a*	*parC1*			X1		156	12	++
H2262	*H*22	B5	D2	TEM-1A	CTX-M-9	*gyrA1a*	*parC1*	*aadB*, *aacA4*, *aadA2*	*qnrA1*, *sul1*, *tet*(A), *dfrA12*	FII, FIB, N, HI2	F2:A−:B1, N-ST9, HI2-ST1	MG828	21	++
005-019	*H*22	B5	D1	TEM-1B		*gyrA1a*	*parC1*	*aadA1*	*sul1*, *tet*(A), *dfrA1*	FII, FIB	F24:A−:B6		16	++
H1088	*H*30	C0	C2	TEM-1B		*gyrA1a*	*parC1a+A199G*	*aadA5*	*mph*(A), *sul1*, *dfrA17*	FII, FIA, FIB	F36:A1:B20	156, BS512, IMGS31	14	−
B1G9	*H*30	C0	C2	TEM-1B		*gyrA1a*	*parC1a*	*aadA5*	*mph*(A), *sul1*, *dfrA17*	FII, FIA, FIB	F22:A1:B20	MG828	15	−
H2214	*H*30	C0	C2			*gyrA1a*	*parC1a*			FII, FIA, FIB	F36:A1:B20	MG828	14	−
B1A5	*H*30	C1	C3	TEM-1B		*gyrA1AB*	*parC1aAB*	*aac(3)-IId*		FII, FIA, FIB	F2:A2:B20	IMGS31	12	−+
B1H12	*H*30	C1	C3	TEM-1B		*gyrA1AB*	*parC1aAB*	*aadA5*, *strA*, *strB*	*mph*(A), *sul1-sul2*, *tet*(A), *dfrA17*	FII, FIA, FIB	F1:A2:B20	156, IMGS31	12	−+
B2B2	*H*30	C1	C2	TEM-1B	CTX-M-14	*gyrA1AB*	*parC1aAB*			FII, FIA, FIB	F10:A2:B20	156, IMGS31	14	−
02	*H*30	C1	C3	TEM-1B		*gyrA1AB*	*parC1aAB*	*aadA5*	*sul1*, *dfrA17*	FII, FIA, FIB, N	F1:A2:B20, N−UST	KPHS6	12	−+
39	*H*30	C1	C2	TEM-1B		*gyrA1AB*	*parC1aAB*	*strA*, *strB*, *aadA5*	*mph*(A), *sul1-sul2*, *tet*(A), *dfrA17*	FII, FIA, FIB	F1:A2:B20	BS512, KPHS6	14	−
183	*H*30	C1	C3	TEM-1B		*gyrA1AB*	*parC1aAB*			FII, FIA, FIB, I1, X1	F1:A2:B20, I1-ST196	BS512, IMGS31	13	−
187	*H*30	C1	C3	TEM-1B		*gyrA1AB*	*parC1aAB*	*strA*, *strB*, *aadA5*	*mph*(A), *sul1-sul2*, *tet*(A), *dfrA17*	FII, FIA, FIB, I1, X4	F1:A2:B20, I1-UST	BS512	12	−+
CES103C	*H*30	C1	C3	TEM-1B		*gyrA1AB*	*parC1aAB*	*aac(3)-IId*		FII, FIA, FIB	F1:A2:B20	BS512	12	−
CES9C	*H*30	C1	C2	TEM-1B		*gyrA1AB*	*parC1aAB*			FII, FIA	F1:A2:B−	IMGS31	14	−
CES164C	*H*30	C1	C2	TEM-1B		*gyrA1AB*	*parC1aAB*			FII, FIA, FIB	F1:A2:B20	BS512	13	−
H3084B	*H*30	C1	ND	TEM-1B		*gyrA1AB*	*parC1aAB*	*aac(3)-IId*		FII FIA FIB, I1, N	F1:A2:B20, I1-UST, N-ST9	BS512	11	−+
C5	*H*30	C1-M27	C2	TEM-1B	CTX-M-27	*gyrA1AB*	*parC1aAB*	*strA*, *strB*, *aadA5*	*mph*(A), *sul1-sul2*, *tet dfrA17*	FII, FIA, FIB, I1	F1:A2:B20, I1-ST193	BS512, IMGS31	14	−
TN03	*H*30	C2	C2	TEM-1B, OXA-1	CTX-M-15	*gyrA1AB*	*parC1aAB*	*aac(3)-IIa*, *aac(6′)Ib-cr*	*tet*(A)	FII, FIA	F2:A1:B−	BS512	14	−
B5B6	*H*30	C2	C2	TEM-1B, OXA-1	CTX-M-15	*gyrA1AB*	*parC1aAB*	*aac(3)-IIa*, *aac(6′)Ib-cr*		FII, FIA	F2:A1:B−	IMGS31	14	−+
B12I1	*H*30	C2	ND			*gyrA1AB*	*parC1aAB*			FII, FIA	F2:A1:B−	BS512, IMGS31	11	−+
C23	*H*30	C2	ND	TEM-1B, OXA-1	CTX-M-15	*gyrA1AB*	*parC1aAB*	*aac(6′)Ib-cr*, *aadA5*	*mph*(A), *sul1*, *tet*(A), *dfrA17*	FII, FIA	F2:A1:B−	BS512	15	−
C3	*H*30	C2	ND	TEM-1B, OXA-1	CTX-M-15	*gyrA1AB*	*parC1aAB*	*aac(6′)Ib-cr*, *aadA5*	*mph*(A), *sul1-sul2*, *tet*(A), *dfrA17*	FII, FIA	F2:A1:B−	BS512	15	−+

a Inc, incompatibility; ND, not described so far; ++, early biofilm producer; −+ delayed biofilm producer; −, never a biofilm producer.

All strains but one (strain 208) harbored an IncF plasmid with an FII, FIA, and FIB replicon allele composition corresponding to the one previously described for clade B and C0/C1 and C2 subclade strains (13), except for one C1 subclade strain (CES9C) that lacked the FIB replicon observed in C2 subclade strains (Table 1). Four clade B strains (two of the B1 subclade, one of the B3 subclade, and one of the B5 subclade) harbored a RepF1C replicon that belonged to the RepFIIA family (18), and one B3 subclade strain harbored two replicons of the RepFIIA family (Table 1).

As shown in Table 1, the 39 strains comprised 30 strains, 10 of which were clade B, displaying diverse antibiotic resistance-encoding genes with a gradual accumulation from clade B to clade C.

### *In vitro* phenotypes of the 39 studied O25b:H4 ST131 strains.

We first assessed four *in vitro* phenotypes of the 39 collected strains: the maximum growth rate (MGR), the kinetics of early biofilm formation, and the expression of both type 1 and curli fimbriae.

**FIG 2:**
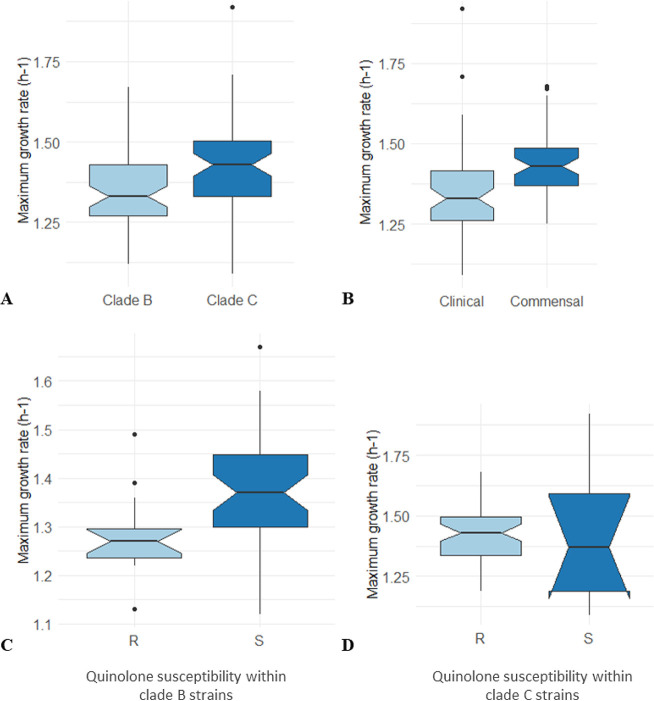
Maximum growth rate (MGR) in LB of the 39 O25b:H4 ST131 Escherichia coli strains studied. MGR was calculated from three independent LB culture assays and is expressed in hours*^−^*^1^. Box plots indicate the MGR distribution across strains, and horizontal black bars indicate median values. The upper and lower ends of the box correspond to the upper and the lower quartiles, respectively. Error bars represent the standard error of the mean from three experiments. Outliers are represented by dots. (A) MGR of clade B and clade C strains. (B) MGR of clinical and commensal (fecal) strains. (C) MGR of nalidixic acid-resistant (R) and -susceptible (S) strains within clade B. (D) MGR of nalidixic acid-resistant (R) and -susceptible (S) strains within clade C. No significant difference was observed in each comparison (Wilcoxon test).

For the MGR measured in a complex medium (lysogeny broth [LB]) under planktonic and shaking conditions (Fig. 2), there was no significant difference regardless of the comparisons made: clade B versus clade C (*P* = 0.08) (Fig. 2A), clinical versus fecal strains (*P* = 0.07) (Fig. 2B), nalidixic acid-resistant versus nalidixic acid-susceptible clade B strains (*P* = 0.9) (Fig. 2C), and nalidixic acid-/ciprofloxacin-resistant versus nalidixic acid-/ciprofloxacin-susceptible clade C strains (*P* = 0.9) (Fig. 2D).

**FIG 3:**
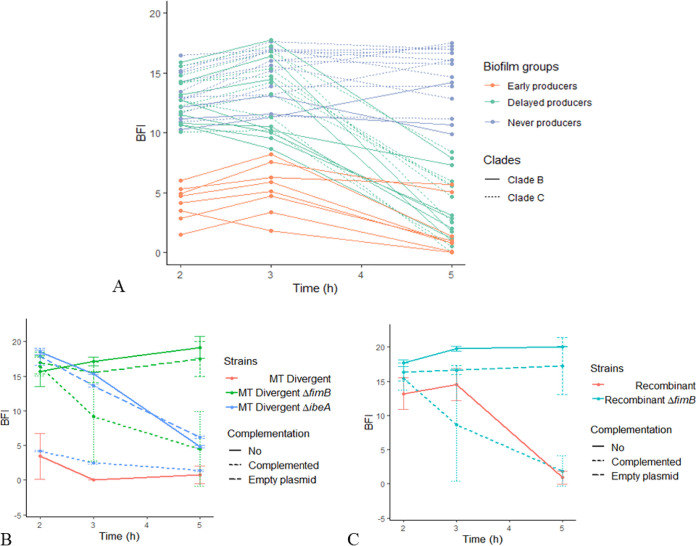
Early biofilm kinetics of the 39 O25b:H4 ST131 Escherichia coli strains and mutants studied. The BioFilm ring test was used to measure early biofilm formation, i.e., after 2, 3, and 5 h of static incubation. Biofilm index (BFI) values are inversely proportional to the biofilm formation capacity, with a BFI value of ≤10 meaning biofilm formation and a BFI value of >10 meaning no biofilm formation. (A) Biofilm kinetics for the 39 O25b ST131 strains. Clade B strains were more frequently early biofilm producers than clade C strains (*P* < 0.0001). (B) Biofilm kinetics for (i) MT Divergent and its Δ*fimB*::FRT and Δ*ibeA*::FRT mutants, which became never and delayed biofilm producers, respectively, in the 5-h assay period; (ii) MT Divergent Δ*fimB*::FTR and Δ*ibeA*::FRT mutants complemented with the wild-type *fimB* gene (Δ*fimB*c) and the wild-type *ibeA* gene (*ibeA*c), both of which were harbored by plasmid pSC-A, in which the early biofilm formation phenotype of the parental strain was partially and totally restored, respectively; and (iii) MT Divergent Δ*fimB*::FTR and MT Divergent Δ*ibeA*::FRT mutants complemented with the empty plasmid pSC-A (Δ*fimB*p and Δ*ibeA*p, respectively). (C) Biofilm kinetics of Recombinant, its Δ*fimB*::FRT mutant, and the Recombinant Δ*fimB*::FRT mutant complemented with the wild-type *fimB* gene harbored by plasmid pSC-A (Δ*fimB*c) or transfected with the empty plasmid pSC-A (Δ*fimB*p). The Δ*fimB*::FRT mutant lost the capacity to produce a biofilm early, whereas when it was complemented with the wild-type *fimB* gene, it produced a biofilm earlier than the parental strain.

We investigated the kinetics of early biofilm formation because previous results had shown that among a large collection of E. coli clinical isolates composed of 99 sequence type (ST) lineages, the O25b:H4 ST131 lineage belongs to the few lineages able to form a biofilm early in the experiment (19). We showed here that clade B strains were more frequently early biofilm producers than clade C strains (*P* < 0.001). More specifically, we found three significantly different phenotypes of early biofilm production (*P* < 0.0001) among the 39 studied strains (Fig. 3A; Table 1): early and persistent producers (biofilm index [BFI] ≤ 10 at 2, 3, and 5 h), delayed producers (BFI > 10 at 2 and 3 h but BFI ≤ 10 at 5 h), and never producers (BFI > 10 at 2, 3, and 5 h). As BFI values after 2 and 3 h of incubation classified a given strain into the same early biofilm formation phenotype (Fig. 3A), only BFI values obtained after 2 and 5 h of incubation were considered for further analyses. The 19 clade B strains were either early producers (*n* = 8), delayed producers (*n* = 8), or never producers (*n* = 3), whereas the 20 clade C strains were either delayed producers (*n* = 8) or never producers (*n* = 12) (Fig. 3A; Table 1). Within clade B, there was a significant difference between the seven B5 subclade strains (early producers, *n* = 5; delayed producers, *n* = 2) and the seven B4 subclade strains (delayed producers, *n* = 4; never producers, *n* = 3) (*P* = 0.02). Inversely, there was no significant difference between the three clade C subclades regarding the early biofilm formation phenotypes.

As the ability to form a biofilm is linked to type 1 and curli fimbria expression (20), we studied the expression of these types of fimbriae in the 39 strains at different times and under different growth conditions. After 2 and 5 h of shaking growth, the early biofilm producers more frequently expressed type 1 fimbriae than the delayed (*P* < 0.01) and never (*P* ≤ 0.0001) producers, and clade B strains more frequently expressed type 1 fimbriae than clade C strains (*P* ≤ 0.0001) (Table 2). All these significant differences were still observed after 24 h of growth under shaking conditions, but no significant difference was observed under static conditions (Table 2). Curli fimbria expression (Table 3), as assessed by the Congo red morphotype, was significantly associated with the early biofilm formation phenotype (*P* < 0.0001). Significantly more clade B strains (47%) than clade C strains (15%) were positive by the Congo red assay (*P* < 0.001).

**TABLE 2:** Yeast agglutination test applied to the 39 O25b:H4 Escherichia coli strains under different growth conditions and at different time points according to biofilm production phenotype and clade type^*a*^

Group	No. (%) of strains positive by yeast agglutination test			
	Early test, shaking culture		Standard test, 24 h^*a*^	
	2 h	5 h	Shaking culture	Static culture
Biofilm ++ (*n* = 8)	8 (100)^*b*^^,^^*c*^	8 (100)^*b*^^,^^*c*^	8 (100)^*b*^^,^^*c*^	8 (100)
Biofilm −+ (*n* = 16)	5 (31)^*b*^^,^^*c*^	5 (31)^*b*^^,^^*c*^^,^^*d*^	4 (25)^*b*^^,^^*c*^	11 (69)
Biofilm − (*n* = 15)	2 (13)^*c*^	0^*c*^^,^^*d*^	0^*c*^	11 (73)
Clade B (*n* = 19)	15 (79)^*c*^	13 (68)^*c*^	12 (63)^*c*^	17 (90)
Clade C (*n* = 20)	0^*c*^	0^*c*^	0^*c*^	13 (65)

a The standard test was performed as described by Totsika et al. (21). Early tests were always negative in static cultures (data not shown). ++, early biofilm producer; −+, delayed biofilm producer; −, never biofilm producer. *P* values were determined by Fisher’s exact test.

b *P* < 0.01.

c *P* < 0.0001.

d *P* < 0.05.

**TABLE 3:** Colony color and morphotype on Congo red agar plates of the 39 O25b:H4 ST131 Escherichia coli strains according to biofilm production phenotype and clade type^*a*^

Group	No. (%) of strains with a positive Congo red test	
	Congo red staining	rdar morphotype
Biofilm ++ (*n* = 8)	8 (100)^*b*^	8 (100)^*b*^
Biofilm −+ (*n* = 16)	1 (6)^*b*^	1 (6)^*b*^
Biofilm − (*n* = 15)	4 (27)^*b*^	3 (20)^*b*^
Clade B (*n* = 19)	9 (47)^*c*^	9 (47)^*c*^
Clade C (*n* = 20)	3 (15)^*c*^	2 (10)^*c*^

a ++, early biofilm producer; −+, delayed biofilm producer; −, never biofilm producer; rdar, red, dry, and rough colonies. *P* values were determined by Fisher’s exact test.

b *P* < 0.0001 for the early biofilm producer group versus both the delayed biofilm producer and never biofilm producer groups.

c *P* < 0.001 for clade B versus clade C.

In sum, the MGR in a complex medium did not differ significantly between clade B and clade C strains, whereas clade B strains had a higher capability than clade C strains to form a biofilm early and express type 1 and curli fimbriae earlier.

### Potential role of the *fimB* and the *ibeA* genes in the different phenotypes of early biofilm formation and type 1 fimbria expression identified among the 39 O25b:H4 ST131 strains studied.

**FIG 4:**
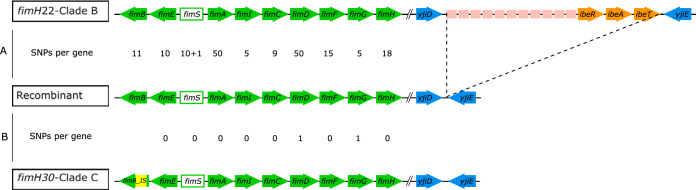
Schematic representation of the *fim* operon and the *gimA* locus in O25b:H4 ST131 *fimH22*-clade B strains, Recombinant, and clade C strains. The genes of the *fim* operon are depicted by green arrows, except for the *fimS* gene, which is an invertible element; those of the *gimA4* operon, including the *ibeART* operon, which are indicated by ocher arrows; and those of the three other operons of the *gimA* locus, which are indicated by pink rectangles. The dashed lines indicate the deletion of the *gimA* locus in both Recombinant and the clade C strains. Yellow indicates the IS*Ec*55 insertion within the *fimB* gene of all clade C strains. (A) Comparison between *fimH22*-clade B strains based on the *fim* operon of a B1 subclade strain (strain S250) and Recombinant (B4 subclade). (B) Comparison between Recombinant and clade C strains based on the operon of a C1 subclade strain (CES164C). SNPs, single nucleotide polymorphisms; +1, the presence of a gap.

To decipher the molecular supports for the different early biofilm formation and type 1 fimbria expression phenotypes observed among the 39 strains, we analyzed the *fim* operon across the 39 studied strains, knowing that (i) the IS*Ec55* insertion sequence, which encodes a cofactor of type 1 fimbria synthesis regulation, has been described within the clade C *fimB* gene (21) and (ii) Recombinant displayed the *fimH30* allele previously thought to be a specific trait of clade C strains. Between the *fim* operon of the *fimH22* clade B strains and that of Recombinant, there were from 5 to 50 SNPs per gene, depending on the gene (Fig. 4). Inversely, between the *fim* operon of Recombinant and that of clade C strains, there was a perfect match, except for two SNPs and the absence of IS*Ec*55 within the *fimB* gene of Recombinant (Fig. 4). We also found a partial deletion of the *fim* operon in two *fimH22* clade B strains, the B4 subclade strain 001-001 and the B5 subclade strain H1447 (data not shown).

To assess the role of the *fimB* gene in early biofilm formation and type 1 fimbria expression, we constructed Δ*fimB* mutants from the B1 subclade strain S250, which displayed an early (from 2 h) phenotype for the two traits (Tables 1 and 4), and Recombinant, which displayed a delayed (at 5 h) phenotype for the two traits (Tables 1 and 4). Both the S250 Δ*fimB* and Recombinant Δ*fimB* mutants lost their ability to form a biofilm (Fig. 3B and C) and to express type 1 fimbriae during the 5-h time period (Table 4). Complementation with the wild-type S250 *fimB* gene rendered the complemented strain a producer of a biofilm from 3 h, but at a level lower than that observed in strain S250 (Fig. 3B), and restored the expression of type 1 fimbriae observed in strain S250 (Table 4). Complementation with the wild-type recombinant *fimB* gene rendered the complemented strain a producer of a biofilm from 3 h, meaning that it was an earlier producer than Recombinant (Fig. 3C), and permitted the expression of type 1 fimbriae during the 5-h period of the experiment instead of only at 3 h, as observed in Recombinant (Table 4). Type 1 fimbria expression was also assessed after 24 h of incubation (Table 4). This experiment showed that the S250 Δ*fimB* mutant did not express type 1 fimbriae at 24 h under either shaking or static conditions, whereas the Recombinant Δ*fimB* mutant and the tested clade C strain (CES164C) did, but only under static conditions (Table 4).

**TABLE 4:** Yeast agglutination test applied to B1 subclade strain S250 (MT Divergent) and its mutants, B4 subclade strain CES131 (Recombinant) and its mutant, and C1 subclade strain CES164 (Emergent)

Strain	Yeast agglutination test result^*a*^			
	Shaking growth			Static growth, standard test at 24 h
	Early test^*b*^		Standard test at 24 h	
	At 2 h	At 5 h		
S250	++++	++++	+++	++++
S250 Δ*fimB*::*kan*	−	−	−	−
S250 Δ*fimB*::FRT/pSC-AS250 *fimB*	++++	++++	+++	+++
S250 Δ*fimB*:: FRT/pSC-A	−	−	−	−
S250 Δ*ibeA*::*kan*	++++	++++	+++	++++
S250 *ΔfimB*::FRT *ΔibeA*::*kan*	−	−	−	−
S250 Δ*ibeART*::*kan*	++++	++++	+++	++++
S250 *ΔfimB*::FRT *ΔibeART*::*kan*	−	−	−	−
CES131C	−	+	−	++++
CES131C Δ*fimB*::*kan*	−	−	−	+++
CES131C Δ*fimB*::FRT/pSC-ACES131C *fimB*	++	+++	+++	++++
CES131C Δ*fimB*::FRT/pSC-A	−	+	−	+++
CES164C	−	−	−	+++
Positive control (E. coli UTI89)	+++	+++	+++	+++
Negative control (E. coli UBA83972)	−	−	−	−

a The yeast agglutination test was positive when agglutination appeared within 3 min. ++++, fast appearance of agglutination with thick aggregations; +++, fast appearance of agglutination with fine aggregations; ++, slow appearance of agglutination with thick aggregations; +, slow appearance of agglutination with fine aggregations; −, no agglutination.

b The early test was always negative under static growth conditions (data not shown).

We then investigated the *ibeA* gene, knowing that (i) it was previously suggested that the brain endothelial cell invasion and adhesion determinants encoded by the *ibeA* gene and the *ibeT* gene, respectively, would have a role in modulating the expression of type 1 fimbriae (22) and (ii) the *ibeA* gene is absent in clade C strains (Table 5). We found that the *gimA* locus, including the *ibeAT* genes, encompassed with the *ibeR* gene in the *gimA4* operon, was present in all clade B strains except for strain 001-001 (data not shown) and Recombinant, in which it was completely deleted, as in clade C strains (Fig. 4). Additionally, we found that the inactivation of the *ibeA* gene of strain S250 rendered the S250 Δ*ibeA* mutant a delayed producer of a biofilm, whereas its complementation with the wild-type *ibeA* gene restored the early biofilm phenotype observed in the parental strain (Fig. 3B). Inversely, *ibeA* inactivation in strain S250, as well as that of the *ibeART* operon, had no impact on the expression of type 1 fimbriae (Table 4).

**TABLE 5:** Main virulence factor-encoding genes in the 39 O25b:H4 ST131 Escherichia coli strains, by clade

Gene	No. (%) of strains			*P* value^*a*^ for clade B vs clade C
	Total (*n* = 39)	Clade B (*n* = 19)	Clade C (*n* = 20)	
Adhesin				
*yfcV*	39 (100)	19	20	
*fimH*	39 (100)	19	20	
*papAH*	2 (5)	2	0	
*papC*	2 (5)	2	0	
*papEF*	3 (8)	2	1	
*sfa* and *foc*	1 (3)	0	1	
*afa* and *dra*	8 (21)	6	2	
Toxin				
*hlyA*	0	0	0	
*hlyF*	10 (26)	10	0	0.0002
*cnf1*	0	0	0	
*cdtB*	7 (18)	7	0	0.003
*sat*	25 (64)	7	18	0.0008
*vat*	0	0	0	
*tsh*	1 (3)	1	0	
Iron uptake				
*iroN*	10 (26)	10	0	0.0002
*fyuA*	39 (100)	19	20	
*iutA*	32 (82)	14	19	
*iucD*	32 (82)	14	19	
*irp2*	39 (100)	19	20	
*chuA*	39 (100)	19	20	
Capsule				
*kpsMII*	33 (85)	19	14	0.02
*kpsMIII*	0	0	0	
*K1*	3 (8)	3	0	
*K2*	0	0	0	
*K5*	30 (77)	16	14	
Miscellaneous				
*iss* type 1	11 (28)	11	0	0.00005
*traT*	36 (92)	17	19	
*cvaC*	5 (13)	5	0	0.02
*ibeA*	17 (44)	17	0	3 × 10^−9^
*usp*	39 (100)	19	20	
*ompT*	39 (100)	19	20	
*malX*	39 (100)	19	20	
				
Mean ± SD VF^*b*^	14.9 ± 2.5	16.6 ± 2.4	13.3 ± 1.3	0.000004

a *P* values (shown when they were <0.05) were obtained by Fisher’s exact test for the gene distribution comparison between clades and by the Wilcoxon rank-sum test for the VF mean comparison.

b VF, virulence factor-encoding gene.

In sum, the targeted-gene analysis performed *in vitro*, i.e., in a simple environment, showed the involvement of both *H*22/*H*30 FimB and IbeA in early biofilm formation and only that of *H*22/*H*30 FimB in the early expression of type 1 fimbriae.

### *In vivo* virulence and ExPEC VF gene content of the 39 studied O25b:H4 ST131 strains.

**FIG 5:**
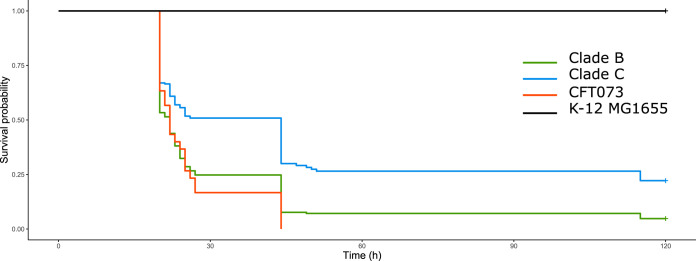
Kaplan-Meier survival curves of mice with sepsis due to the 39 O25b:H4 ST131 Escherichia coli strains studied, by clade. Clade B and C strains and controls (positive control, strain CFT073; negative control, strain K-12 MG1655) are represented by different colored lines. Clade B strains (210 mice) killed mice significantly faster than clade C strains (230 mice) (*P* = 2e−10).

**FIG 6:**
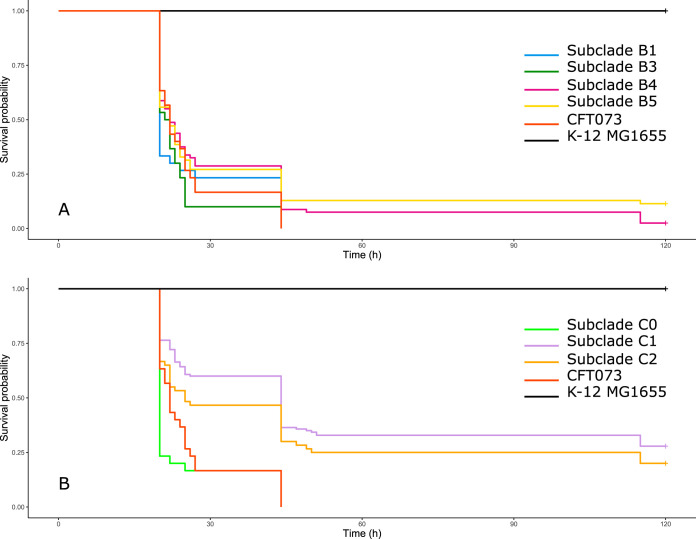
Kaplan-Meier survival curves of mice with sepsis due to the 39 O25b:H4 ST131 Escherichia coli strains studied, by subclade. B subclade strains (A), C subclade strains (B), and controls (positive control, strain CFT073; negative control, strain K-12 MG1655) are represented by different colored lines. No significant difference was observed between B1 subclade strains (30 mice) and those of the B3 subclade (30 mice), B4 subclade (80 mice), and B5 subclade (70 mice), whereas B3 subclade strains killed mice significantly faster than those of the B4 subclade (*P* = 0.02). Among the clade C strains, C0 subclade strains (30 mice) killed mice significantly faster than those of the C1 subclade (140 mice) (*P* = 5e−09) and C2 subclade (60 mice) (*P* = 6e−05), while there was no significant difference between the C1 and C2 subclade strains.

By using a well-calibrated mouse sepsis model (23), we assessed, *in vivo*, a more complex phenotype i.e., the intrinsic extraintestinal virulence, of the 39 strains studied. We found that clade B strains were significantly more virulent than clade C strains (*P* = 2e−10) (Fig. 5). Among the clade B strains (Fig. 6A), no significant difference was observed between the strains of each subclade except for B3 subclade strains, which killed mice significantly faster than those of B4 subclade (*P* = 0.02). Among the clade C strains (Fig. 6B), the C0 subclade strains were significantly more virulent than those of the C1 subclade (*P* = 5e−09) and C2 subclade (*P* = 6e−05), while there was no significant difference between those of the C1 and C2 subclades.

Considering the described association between virulence assessed in mouse sepsis models and the number of genes encoding ExPEC virulence factors (VFs) (24, 25), we analyzed the distribution of the ExPEC VF-encoding genes in the 39 strains. The average number of VF-encoding genes was higher in clade B strains (16.2 ± 2.4) than in clade C strains (13.3 ± 1.3) (*P* = 4e−07) (Table 5). The *hlyF*, *cdtB*, *iroN*, *kpsMII*, *cvaC*, *iss*, and *ibeA* genes were significantly more frequent among clade B strains than among clade C strains, whereas the opposite was found for the *sat* gene (Table 5). Within clade B, there was no significant difference between the mean number of VF genes of the strains of the different B subclades, except for those belonging to the B3 subclade, which displayed a higher mean number of VF genes than those belonging to the B4 subclade (*P* < 0.05) (data not shown). Within clade C, there was no significant difference either between the C1 and C2 subclade strains or between those of the C0 and C2 subclades, but there was a significant difference between those of C0 and C1 subclades, with the C0 subclade strains having a higher mean number of VF genes (*P* = 0.03) (data not shown). Among the four main VF-based virotypes (virotypes A, B, C and D) previously described for O25b:H4 ST131 (26), we found that virotypes D and C were significantly associated with clade B and clade C strains, respectively (*P* < 0.00001), and that two B4 subclade strains (including Recombinant), one C1 subclade strain, and three C2 subclade strains displayed previously undescribed virotypes (Table 1).

In sum, clade B strains displayed a higher *in vivo* virulence than clade C strains in a mouse sepsis model in relation to a greater number of VFs.

### Selection of strains for further *in vivo* experiments.

To go deeper into the differences observed in the *in vitro* and *in vivo* virulence phenotypes between clades B and C, we selected three strains that allowed us to test (i) gene inactivation and (ii) other relevant *in vivo* mouse model complex phenotypes. Thus, within clade B, we selected strain S250, belonging to the B1 subclade, whose fitness had already been assessed in Caenorhabditis elegans and zebrafish models (27) and *in vitro* (28) and which corresponded, in our collection, to one of the two most anciently diverged strains. We called this strain “MT Divergent,” where MT stands for “the most.” Among the C1 and C2 subclade strains that did not show significant differences with regard to early biofilm formation, the mean number of VF genes, and mouse lethality, we selected C1 subclade strain CES164C, which we called “Emergent.” We also retained Recombinant. Table 6 presents the major characteristics displayed by these three strains. To assess *in vivo* the potential role of the *fimB* gene and the *ibeART* operon, we retained the Δ*fimB*::*kan*, Δ*ibeART*::*kan*, and Δ*fimB*::FLP recombination target (FRT) Δ*ibeART*::*kan* mutants of MT Divergent and the Δ*fimB*::*kan* mutant of Recombinant.

**TABLE 6:** Comparative characteristics of the three O25b:H4 Escherichia coli strains selected for competition assays^*a*^

Strain	MGR (h^−1^)	Colicin/phage production	Biofilm formation phenotype	Time (h) of type 1 fimbria expression	Congo red assay phenotype	Antibiotic resistance-encoding genes	No. of VF-encoding genes^*b*^	No. of ExPEC VF-encoding genes	*fim* operon type	*gimA* locus
MT Divergent (B1 subclade S250)	1.35 ± 0.06	No	Early	2^*c*^	rdar		53	14	*H*22	+
Emergent (C1 subclade CES164C)	1.48 ± 0.06	No	Never	24^*d*^	Red staining	*bla*_TEM_, *gyrA1AB*, *parC1aAB*	52	13	*H*30	−
Recombinant (B4 subclade CES131C)	1.47 ± 0.09	No	Delayed	5^*d*^	White staining	*bla*_TEM_, *aac(3)-IId*, *aadA2*, *mph*(A), *sul1*, *tet*(A), *dfrA12*	63	15	*H*30	−

a VF, virulence factor; rdar, red, dry, and rough; +, present.

b See Fig. S2 in the supplemental material for details.

c With shaking growth.

d With static growth.

### Competition assays in mouse models of sepsis, intestinal colonization, and urinary tract infection (UTI).

Before performing competition assays between MT Divergent, Emergent, and Recombinant and between parental strains and mutants in the mouse sepsis model, we checked their virulence phenotype in this model in a monoinfection (Fig. S3). MT Divergent killed mice faster than Emergent (*P* = 0.04) but not than Recombinant (*P* = 0.8), whereas Recombinant and Emergent did not kill mice differently (*P* = 0.06) (Fig. S3A). There was no significant difference either between MT Divergent and its Δ*fimB*::*kan*, Δ*ibeART*:*:kan*, and Δ*fimB*::FRT Δ*ibeART*::*kan* mutants or between Recombinant and its Δ*fimB*::*kan* mutant (Fig. S3B).

**FIG 7:**
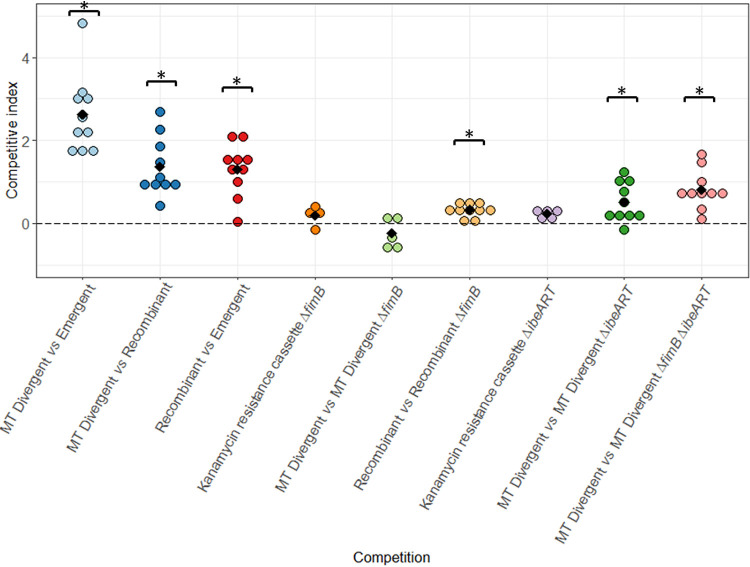
Competitions between MT Divergent, Recombinant, and Emergent and between MT Divergent and its mutants and Recombinant and its mutant in the mouse sepsis model. Competitions were determined by the spleen bacterial load. The competitive index (CI) is expressed in log. A CI above 0 means that the isolate cited first in the legend outcompetes the one cited second, and a CI below 0 means that the isolate cited second outcompetes the one cited first. Each dot represents one mouse, and different colors were attributed to the different competitions. Black diamonds depict the mean values of CI. MT Divergent outcompeted Emergent (*P* = 0.002) and Recombinant (*P* = 0.002), and Recombinant outcompeted Emergent (*P* = 0.002). Before performing competitions between the parental strains and their mutant(s), we checked that the kanamycin resistance cassette introduced in the targeted gene did not have any cost: kanamycin resistance cassette Δ*fimB* (competition between MT Divergent Δ*fimB*::FRT versus MT Divergent Δ*fimB*::*kan* and Recombinant Δ*fimB*::FRT versus Recombinant Δ*fimB*::*kan*, *P* = 0.2) and kanamycin resistance cassette Δ*ibeART* (competition between MT Divergent Δ*ibeART*::FRT versus MT Divergent Δ*ibeART*::*kan*: and MT Divergent Δ*fim*::FRT Δ*ibeART*::FRT versus MT Divergent Δ*fimB*::FRT Δ*ibeART*::*kan*, *P* = 0.062). *, significant difference generated by the Wilcoxon signed-rank test. MT Divergent did not outcompete MT Divergent Δ*fimB*::*kan* (*P* = 0.3), whereas Recombinant outcompeted Recombinant Δ*fimB*::*kan* (*P* = 0.004). MT Divergent outcompeted both MT Divergent Δ*ibeART*::*kan* (*P* = 0.001) and MT Divergent Δ*fimB*::FRT Δ*ibeART*::*kan* (*P* = 0.002).

In competition assays performed in the mouse sepsis model, MT Divergent outcompeted both Recombinant (*P* = 0.002) and Emergent (*P* = 0.002) and Recombinant outcompeted Emergent (*P* = 0.002) (Fig. 7). MT Divergent did not outcompete its Δ*fimB*::*kan* mutant, whereas Recombinant outcompeted its Δ*fimB*::*kan* mutant (*P* = 0.004) (Fig. 7). Inversely, MT Divergent outcompeted its Δ*ibeART*::*kan* (*P* = 0.01) and Δ*fimB*::FRT Δ*ibeART*::*kan* (*P* = 0.002) mutants (Fig. 7). Neither the kanamycin resistance cassette Δ*fimB* (*P* = 0.3) nor the kanamycin resistance cassette Δ*ibeART* (*P* = 0.06) had a cost to *in vivo* virulence in this mouse model (Fig. 7).

**FIG 8:**
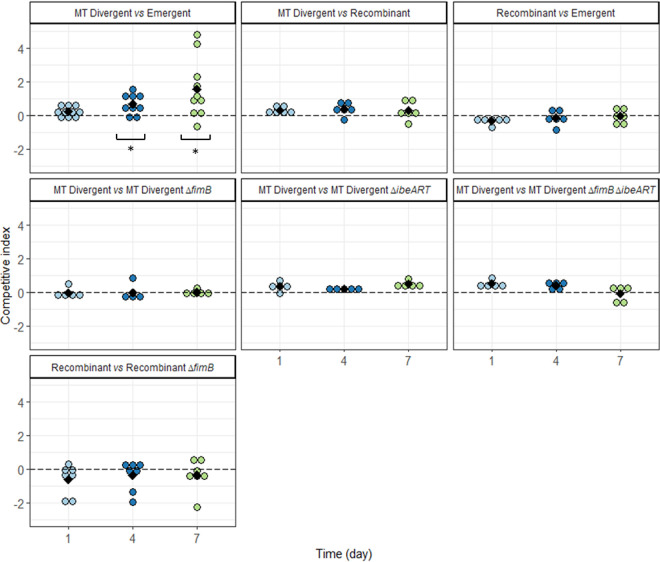
Competitions between MT Divergent, Recombinant, and Emergent and between MT Divergent and its mutants and Recombinant and its mutant in the mouse gut colonization model. Competitions were determined by the fecal bacterial load. The competitive index (CI) is expressed in log. A CI above 0 means that the isolate cited first in the legend outcompetes the one cited second, and a CI below 0 means that the isolate cited second outcompetes the one cited first. Each dot represents one mouse, with the different colors indicating the time period, as follows: light blue, day 1 postinoculation; dark blue, day 4 postinoculation; green, day 7 postinoculation. Black diamonds depict the mean values of CI. All mutants harbored the *kan* gene of the kanamycin resistance cassette. *, significant difference generated by the Wilcoxon signed-rank test and corrected for multiple comparisons by the Benjamini-Hochberg procedure. MT Divergent outcompeted Emergent at both day 4 (*P* = 0.01) and day 7 (*P* = 0.001), whereas the other competitions were not significant (*P* values, between 1 and 0.09, according to the two strain types in the competition and the competition day).

Competitions between the three strains as well as between the parental strains and their mutants were also performed in a mouse intestinal colonization model because intestinal colonization is the first step of infection and extraintestinal virulence is a coincidental by-product of commensalism in B2 phylogenetic group E. coli strains (29). Given that this model requires competitive strains with an equal level of susceptibility to streptomycin and that both MT Divergent and Emergent were susceptible to streptomycin, we used a kanamycin resistance cassette to inactivate the plasmid-mediated *aadA2* gene, encoding streptomycin resistance, in Recombinant. This cassette showed no fitness cost through a competition assay between Recombinant and Recombinant Δ*aadA2*::*kan* under planktonic conditions in LB (data not shown). In this mouse model, we found that MT Divergent outcompeted Emergent (*P* = 0.02) at day 4 and day 7, whereas there was no significant difference at any time point between MT Divergent and Recombinant or between Recombinant and Emergent (Fig. 8). There was also no significant difference between the mutants and their parental strains, i.e., the Δ*fimB*::*kan*, Δ*ibeART*::*kan*, and Δ*fimB*::FRT Δ*ibeART*::*kan* mutants for MT Divergent and the Δ*fimB*::*kan* mutant for Recombinant (Fig. 8).

**FIG 9:**
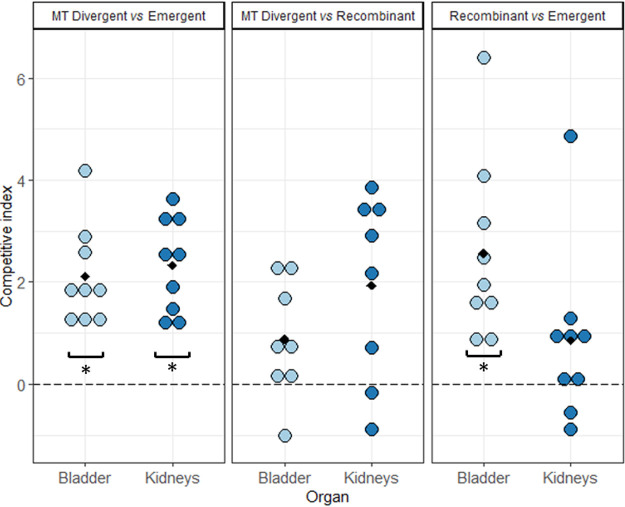
Competitions between MT Divergent, Recombinant, and Emergent in the mouse urinary tract infection model. Competitions were determined by the bladder and kidney bacterial loads. Each dot represents one mouse, with the different colors indicating the organs tested; light blue for urine and dark blues for the kidneys. The competitive index (CI) is expressed in log. A CI above 0 means that the isolate cited first in the legend outcompetes the one cited second, and a CI below 0 means that the isolate cited second outcompetes the one cited first. Black diamonds depict the mean values of CI. *, significant difference generated by the Wilcoxon signed-rank test. MT Divergent outcompeted Emergent in the bladder (*P* = 0.004) and the kidneys (*P* = 0.004), whereas it did not outcompete Recombinant either in the bladder (*P* = 0.08) or in the kidneys (*P* = 0.05). Recombinant outcompeted Emergent in the bladder (*P* = 0.004) but not in the kidneys (*P* = 0.13).

Finally, we put MT Divergent, Recombinant, and Emergent in competition in the mouse UTI model, knowing that clade C strains have frequently been identified among uropathogenic E. coli (UPEC) strains (7). We found that MT Divergent outcompeted Emergent in the bladder (*P* = 0.004) and the kidneys (*P* = 0.004) and that Recombinant outcompeted Emergent in the bladder (*P* = 0.008) but not in the kidneys (*P* = 0.13), whereas there was no significant difference between MT Divergent and Recombinant either in the bladder (*P* = 0.08) or in the kidneys (*P* = 0.05) (Fig. 9). In sum, MT Divergent outcompeted Emergent regardless of the mouse model used.

## DISCUSSION

To find out the factors other than multidrug resistance that could have favored the dissemination of E. coli O25b:H4 ST131 clade C, we used an original approach based on phenotypic comparisons of clade B and clade C strains. To this end, we established an O25b:H4 ST131 collection composed of clade B (*n* = 19) and clade C (*n* = 20) strains belonging to the various B and C subclades. However, in this collection, we found a B4 subclade strain with a *fimH30* allele (called “Recombinant”), a peculiar feature previously found in another B4 subclade strain (5). Here, we clarified that Recombinant shared with clade C not only the same *fim* operon (however, it lacked the IS*Ec55* sequence always found in the clade C *fimB* gene) but also a deletion of the *gimA* locus, including the *ibeART* operon. This suggests that identical genetic events had occurred in clades B and C but, apparently, with a lower adaptive success in the genetic background of clade B than in that of clade C.

In all but one (MGR in LB) phenotypic experiment applied to the 39 strains, we found that clade B had a significantly higher virulence and competitive ability than clade C, and the level of these features for Recombinant was intermediate between those of *fimH22*-clade B and clade C strains. This indicated that, despite the same ability to grow under optimal conditions *in vitro*, the strains behaved differently in more complex environments more closely related to the normal niches of E. coli ST131.

We confirmed that the early biofilm production phenotype, which is a property displayed by <10% of ExPEC strains belonging to few E. coli lineages (19), was always displayed by clade B strains and never by clade C strains (19, 28). By using the MT Divergent Δ*H*22-*fimB* and Recombinant Δ*H*30-*fimB* mutants, we showed that biofilm formation and type 1 fimbria expression during the first 5 h of incubation require a functional *fimB* gene. We also showed that the Recombinant *H*30-Δ*fimB* mutant required, like Emergent, at least 24 h of growth under static conditions to express type 1 fimbriae, whereas the MT Divergent Δ*H*22-*fimB* mutant was unable to express these fimbriae at 24 h under both shaking and static conditions. All these findings strongly suggest that the presence of the IS*Ec55* sequence in the *fimB* gene of clade C strains contributes to their incapacity to form a biofilm early and express type 1 fimbriae before 24 h of incubation and that, in addition to the genetic differences between the two *fim* operon types, there could be differences in their regulation. Our results for type 1 fimbria expression by Emergent and other clade C strains are concordant with those found by Totsika et al. with clade C strain EC958 and other O25b:H4 ST131 strains harboring the IS*Ec55* sequence in the *fimB* gene (21). Using a mouse UTI model, these authors showed that strain EC958 colonized the bladder when type 1 fimbria expression was strong and persisted in urine when it was weak (21). On the other hand, Gunther et al., also through competition assays in a mouse UTI model between a wild-type CFT073 strain and mutants expressing their type 1 fimbriae only weakly or strongly, concluded that type 1 fimbria phase variation contributes significantly to virulence early (24 h postinoculation) in the 76-h course of the mouse UTI and profoundly influences colonization of the bladder (30). Our competition assays, performed in the same mouse UTI model as that used in the cited studies, showed that MT Divergent outcompeted Emergent in the bladder and the kidneys. These *in vivo* results, combined with those that we obtained *in vitro*, strongly suggest that the early expression of type 1 fimbriae allows clade B to quickly cause bladder colonization and pyelonephritis, while clade C requires more time to have a functional temporal on/off regulation of type 1 fimbria expression in a link to bladder colonization and urine persistence, respectively, as described for multiple UPEC strains by Schwan and Ding (31). In short, the loss of early expression of type 1 fimbriae by clade C strains makes them classical phylogroup B2 UPEC strains with, however, a peculiar trait, i.e., antibiotic and even multiantibiotic resistance (32).

The other major significant phenotypic differences between clade B and clade C consisted of a higher VF score and faster killing of mice by clade B than by clade C. This result is in accordance with the results of previous studies that showed the association between virulence, as assessed in mouse sepsis models, and the number of VFs (24, 25). Within clade B, B3 subclade strains had both higher VF scores and a higher *in vivo* virulence than B4 subclade strains, whereas within clade C, C0 subclade strains had higher VF scores and a higher *in vivo* virulence than either C1 or C2 subclade strains, but this difference was not found between C1 and C2 subclade strains. Making comparisons between our results and those previously obtained with the same mouse sepsis model is difficult because the previous studies used only surrogates of clade B and clade C strains (strains with susceptibility to fluroquinolones, *fimH30* versus non-*fimH30* alleles, and virotypes) (33–35). Using well-characterized strains, as we did, permitted the various levels of *in vivo* virulence between and within the clades and subclades of O25b:H4 ST131 to be deciphered.

We performed a genome-wide association study (GWAS) using Scoary software (36), which assesses the association between the presence/absence of genes and *in vivo* virulence in the mouse sepsis assay, but did not obtained any significant hit over the 17,951 tested genes (data not shown). This failure could have been due to the small number of strains tested; the limited genetic variability of the strains, all of which belonged to a single E. coli lineage (37); or an *in vivo* virulence resulting from VF cooperation in an additive manner to achieve extraintestinal virulence (25). However, targeted-gene analyses performed in competition assays in the mouse sepsis model showed that the *H*30-*fimB* gene was associated with the *in vivo* virulence of Recombinant and the *ibeART* operon was associated with that of MT Divergent. These results, together with those showing that MT Divergent outcompeted both Recombinant and Emergent and that Recombinant outcompeted Emergent in the mouse sepsis model, strongly suggest that the absence of the *ibeART* operon in Recombinant and clade C and the nonfunctionality of the *H*30-*fimB* gene in clade C may have contributed to the gradually lower levels of *in vivo* virulence displayed by Recombinant and Emergent in the mouse sepsis model.

As competition assays provide more evidence than assays with single strains of the difference in fitness of only a few percentage points, indicating stronger differences, we used only competition assays to study more thoroughly three representative strains (i.e., MT Divergent, Recombinant, and Emergent) in a mouse model of sepsis, gut colonization, and UTI. In all competition assays, Emergent was outcompeted by MT Divergent regardless of the mouse ecological niches, whereas it was outcompeted by Recombinant, depending on the niches: it was outcompeted in the blood and partially outcompeted in the urinary tract (in the bladder but not in the kidneys) but was not outcompeted in the gut. Such features may suggest an adaptation to ecological niches within the ST131 lineage, as assessed by McNally et al. (14).

In conclusion, by measuring several simple and more complex phenotypes, we highlighted the lower level of virulence of clade C strains than of clade B strains, what had previously been suggested in the zebrafish model (27).

It may appear to be surprising that the clade most disseminated worldwide, i.e., ST131 clade C, displays attenuated virulence. We propose two explanations for its widespread dissemination. First, the resistance to fluoroquinolones displayed by clade C may come at a direct cost (38). This cost would not be revealed in the MGR under planktonic conditions but would manifest *in vivo*, as suggested here in various mouse models. In spite of this cost, antibiotic resistance allowed clade C to spread. Second, the lower virulence may actually confer an additional evolutionary advantage to clade C. According to the trade-off theory (39), host exploitation by a pathogen evolves to an optimal level under a balance between the benefits in terms of transmission and the costs in terms of host mortality (40). Thus, lower virulence could confer improved fitness overall by avoiding severe infections (41, 42), even if it comes at the cost of a lower colonization ability.

Whether the phenotypic clade B and clade C differences represent a cost, in spite of which clade C is successful, or an additional adaptation remains an open question that requires further work, especially as the *in vivo* behavior of fluoroquinolone-susceptible Recombinant favors the additional adaptation hypothesis.

## MATERIALS AND METHODS

More technical details for each section of Materials and Methods are available in Text S1 in the supplemental material.

### Bacterial strains.

The 39 O25b:H4 ST131 E. coli strains studied comprised 18 *fimH22* and 21 *fimH30* strains obtained between 1993 and 2012 from different geographic origins and sources (Table S1). The E. coli CFT073 and E. coli K-12 MG1655 strains were used as positive and negative controls, respectively, in the mouse sepsis model, and the E. coli UTI89 and E. coli UBA83972 strains were used as positive and negative controls, respectively, in the yeast agglutination assays.

### Genome sequencing and analysis.

Whole-genome sequencing (WGS) of the 39 studied strains was performed (Table S2). All genomes were analyzed for plasmid content and typing (with the Plasmid Finder tool, which showed an identity of >95%, and plasmid multilocus sequence typing [pMLST] [43]). The Abricate tool (44) was used to detect (i) genes encoding antibiotic resistance with the ResFinder program (45) and (ii) genes encoding VF with a custom virulence database composed of VirulenceFinder (46), the virulence factor database (VFDB) (47), and the classical ExPEC VF-encoding genes (19). Virotypes were determined as previously described (26). All contigs were submitted to the MicroScope platform (48) for further gene investigations, such as the investigation of the *gyrA* and *parC* genes. When necessary, the presence or absence of some genes was controlled by PCR.

To assess the different B and C subclades among the 39 collected strains as well as among the 218 bacteremia O25b ST131 E. coli strains collected by Kallonen et al. in England between 2001 and 2012 (9), we complemented the genomes of these 257 strains with those of 21 strains representing the genetic diversity of the B and C subclades (5) and constructed a phylogenetic tree from nonrecombinant SNPs of the core genome genes using the maximum likelihood method. To infer the linear trend of the isolates of Kallonen et al. (9) from 2001 to 2012, we fitted a logistic model to the presence/absence of each subclade as a function of time (in years).

### Gene deletion and complementation.

Replacement by a kanamycin resistance cassette was used to inactivate genes following a strategy adapted from that of Datsenko and Wanner (49). When necessary, the kanamycin resistance-encoding gene of the cassette was eliminated, as previously described (49). Δ*fimB*::FRT and Δ*ibe*A::FRT mutants were complemented with the parental wild-type gene cloned into pSC-A-amp/kan by using a StrataClone PCR cloning kit (Agilent Technologies, Massy, France). The primers and plasmids used are listed in Tables S3 and S4.

### Kinetics of early biofilm formation.

The primary step (5 h of incubation) of biofilm formation was measured using the BioFilm ring test (BioFilm Control, Saint-Beauzire, France) according to the manufacturer’s recommendations and as previously described (19, 28). The BFI, which had values ranging from 20 (the absence of biofilm formation) to 0 (high level of biofilm formation), is inversely proportional to the biofilm formation ability. A BFI value of 10 was chosen as the biofilm production cutoff (a BFI of ≤10 indicates biofilm formation, a BFI of >10 indicates no biofilm formation).

### Expression of type 1 fimbriae.

Expression of type 1 fimbriae was assessed by using the yeast (Saccharomyces cerevisiae) cell agglutination assay, as previously described (21), and, after adaptations to highlight the early expression of type 1 fimbriae, by using 10 μl of a pellet obtained after centrifugation (3,000 × *g*, 10 min) of 3 ml of LB culture after 2- and 5-h incubations. This test was positive when aggregations appeared within 3 min.

### Curli expression and cellulose production.

Congo red assays were used to assess curli fimbria expression and cellulose production, as previously described (50). Assay positivity resulted in the red, dry, and rough (rdar) colony morphotype.

### Colicin and/or phage production.

Colicin and/or phage production in the strains tested was detected as previously described (51) in competition assays.

### Maximum growth rate.

The maximum growth rate (MGR) assay was performed as previously described (52) in LB by using an automatic spectrophotometer (Tecan Infinite F200 Pro) that measures the optical density at 600 nm in each well every 5 min over a period of 24 h. The experiment was repeated three times. Growth curves were then analyzed, and the MGR was calculated and expressed in hours^−1^.

### Mouse models.

**(i) Monoinfection assay in the sepsis model.** The individual ability of the 39 strains and mutants to cause mouse sepsis was assessed as previously described (53). From 10 to 20 mice were used for each assay. Kaplan-Meier curves of mouse survival were performed.

**(ii) Competition assays in sepsis, intestinal colonization, and UTI models.** In competition assays, the relative ability of the two strains tested together (i) to cause sepsis was determined from the spleen bacterial load (53), (ii) their relative ability to colonize the intestine mouse was determined from the fecal bacterial load at days 1, 4, and 7 postinoculation (52), and (iii) their relative ability to cause an ascending unobstructed UTI was determined from the bladder and kidney bacterial load (54). From 5 to 10 mice were used for each competition assay. Competitive indexes (CI) were obtained using the following formula: [(log number of CFU of isolate1/log number of CFU of isolate2) at *T_x_*]/[(log number of CFU of isolate1/log number of CFU of isolate2) at *T*_0_], with isolate1 being the first strain cited in the figure legends, isolate2 being the second one cited in the figure legends, *T_x_* being time *x*, and *T*_0_ being time zero.

### Statistical analysis.

The Wilcoxon rank-sum test was used to compare the average number of VF-encoding genes per clade, and Fisher’s exact test was used to test the distribution of each VF-encoding gene between the clades. The individual fitness measurement was estimated by used of a mixed model with the random effect on the strain to take into account the triplicate determination. For biofilm data, a two-way repeated-measures analysis of variance, followed by Tukey’s range test, when there were three groups, was performed. The association between groups (biofilm phenotypes, yeast agglutination test, Congo red assay, and virotype assay) was assessed by Fisher’s exact test. For *in vivo* competitions, a nonparametric Wilcoxon test on paired data was conducted on CI values, and *P* values were corrected for multiple comparisons by the Benjamini-Hochberg procedure (55), when necessary. Mouse survival differences were determined by the log-rank test. A significance level of <0.05 was used for all tests. All statistical analyses were carried out with R software (56).

### Ethics statement.

Murine protocols of sepsis, intestinal colonization, and pyelonephritis (protocol numbers APAFIS#4948- and APAFIS#4951-2016020515004032 v2, 2016021216251548 v4, and APAFIS#4950-2016021211417682 v4, respectively) were approved by the French Ministry of Research and by the Ethical Committee for Animal Experiments, CEEA-121, Comité d’éthique Paris-Nord.

### Data availability.

The raw sequences of the 39 isolates were deposited in GenBank under BioProject accession numbers PRJNA320043 and PRJNA566165.

## Supplementary Material

Supplemental file 1

Supplemental file 2

Supplemental file 3

Supplemental file 4

Supplemental file 5
