# Supplementary material for: Success of Escherichia coli O25b:H4 Sequence Type 131 Clade C Associated with a Decrease in Virulence
Source: Infect Immun. 2020 Nov 16;88(12):e00576-20. doi: 10.1128/IAI.00576-20 (PMC7671891; doi:10.1128/IAI.00576-20)
Supplement: Supplemental file 1 [file IAI.00576-20-s0001.pdf]

Success of *Escherichia coli* O25b:H4 ST131 clade C associated with a decrease in virulence

Marion DUPRILOT[a,b‡], Alexandra BARON[a‡], François BLANQUART[a,c], Sara DION[a], Cassandra POUGET[d], Philippe LETTÉRON[e], Saskia-Camille FLAMENT-SIMON[f], Olivier CLERMONT[a], Erick DENAMUR[a,g], Marie-Hélène NICOLAS-CHANOINE[a]#

[a] Université de Paris, INSERM, IAME, 75018 Paris, France

[b] AP-HP, Laboratoire de Microbiologie, Hôpital Beaujon, 92110 Clichy, France

[c] Centre for Interdisciplinary Research in Biology (CIRB), Collège de France, CNRS, INSERM, PSL Research University, 75005 Paris, France

[d] VBMI, INSERM U1047, Université de Montpellier, Nîmes, France

[e] Université de Paris UMR 1149 INSERM-ERL CNRS 8252, 75018 Paris, France

[f] Laboratorio de Referencia de *Escherichia coli* (LREC), Departamento de Microbioloxía e Parasitoloxía, Facultade de Veterinaria, Universidade de Santiago de Compostela (USC), Lugo, Spain.

[g] AP-HP, Laboratoire de Génétique Moléculaire, Hôpital Bichat, 75018 Paris, France

[‡]MD and AB share first authorship on this work

#Address correspondence to:

Marie-Hélène NICOLAS-CHANOINE, e-mail: marie-helene.nicolas-chanoine@inserm.fr

Running Head: Loss of virulence in the globally emergent ST131 clade C

Table S1. Characteristics of the 39 O25b:H4 ST131 *Escherichia coli* strains known at collection time

Strain	Origin	Year	Source	O type	Phylo-group	*fimH*	Susceptibility		ESBL	Reference
							Nalidixic acid	Ciprofloxacin		
B11J12	France	2005	Blood	O25b	B2 sg1	*H*22	R	S	/	(1)
S250	France	2006	Feces	O25b	B2 sg1	*H*22	S	S	/	(2–5)
CES106C2	France	2007	Feces	O25b	B2 sg1	*H*22	S	S	+	(1)
H2381	Spain	2008	Blood	O25b	B2 sg1	*H*22	R	S	/	(6)
H3345	Spain	2011	Blood	O25b	B2 sg1	*H*22	R	S	+	This study
H1659	Spain	2005	Blood	O25b	B2 sg1	*H*22	S	S	/	(6, 7)
B7J19	France	2005	Blood	O25b	B2 sg1	*H*22	S	S	/	(1)
B12C27	France	2005	Blood	O25b	B2 sg1	*H*22	S	S	/	(1)
B5E11	France	2006	Blood	O25b	B2 sg1	*H*22	S	S	/	(1)
011-005	France	2009	Feces	O25b	B2 sg1	*H*22	S	S	/	(1)
001-001	France	2010	Feces	O25b	B2 sg1	*H*22	S	S	/	(1)
H219B	Spain	1993	Blood	O25b	B2 sg1	*H*22	S	S	/	(1)
H1447	Spain	2004	Blood	O25b	B2 sg1	*H*22	R	S	/	(6)
H1698	Spain	2005	Blood	O25b	B2 sg1	*H*22	S	S	/	(6, 7)
196	France	2006	Feces	O25b	B2 sg1	*H*22	S	S	/	This study
208	France	2006	Feces	O25b	B2 sg1	*H*22	S	S	/	(2, 5)
H2262	Spain	2008	Blood	O25b	B2 sg1	*H*22	S	S	+	(2, 5)
005-019	France	2009	Feces	O25b	B2 sg1	*H*22	S	S	/	This study
CES131C	France	2008	Feces	O25b	B2 sg1	*H*30	S	S	/	(1)
H1088	Spain	2001	Blood	O25b	B2 sg1	*H*30	S	S	/	This study
B1G9	France	2005	Blood	O25b	B2 sg1	*H*30	S	S	/	(1)
H2214	Spain	2007	Blood	O25b	B2 sg1	*H*30	S	S	/	This study
B1A5	France	2005	Blood	O25b	B2 sg1	*H*30	R	R	/	(1)
B1H12	France	2005	Blood	O25b	B2 sg1	*H*30	R	R	/	(1)
B2B2	France	2005	Blood	O25b	B2 sg1	*H*30	R	R	+	(1)
02	France	2006	Feces	O25b	B2 sg1	*H*30	R	R	/	(2, 5)
39	France	2006	Feces	O25b	B2 sg1	*H*30	R	R	/	(2, 5)
183	France	2006	Feces	O25b	B2 sg1	*H*30	R	R	/	(2, 5)
187	France	2006	Feces	O25b	B2 sg1	*H*30	R	R	/	(2, 5)
CES103C	France	2007	Feces	O25b	B2 sg1	*H*30	R	R	/	(1)
CES9C	France	2007	Feces	O25b	B2 sg1	*H*30	R	R	/	(1)
CES164C	France	2009	Feces	O25b	B2 sg1	*H*30	R	R	/	(1)
H3084B	Spain	2010	Blood	O25b	B2 sg1	*H*30	R	R	/	This study
C5	France	2012	Feces	O25b	B2 sg1	*H*30	R	R	+	(8)
TN03	France	2002	Urine	O25b	B2 sg1	*H*30	R	R	+	(9)
B5B6	France	2005	Blood	O25b	B2 sg1	*H*30	R	R	+	(1)
B12I1	France	2005	Blood	O25b	B2 sg1	*H*30	R	R	/	(1)
C23	France	2012	Feces	O25b	B2 sg1	*H*30	R	R	+	(8)
C3	France	2012	Feces	O25b	B2 sg1	*H*30	R	R	+	(8)

S: susceptible; R: resistant; ESBL: extended-spectrum β-lactamase

Table S2. Genome sequence quality

Strain	Total length	Contigs number ≥ 200 bp	N50	Coverage
B11J12	5023916	88	397655	171
S250	4996154	46	363831	319
CES106C2	5201144	184	165664	190
H2381	5101792	122	319264	216
H3345	5276781	178	152436	182
H1659	5119271	92	156664	361
B7J19	5197897	104	190998	624
B12C27	5132487	84	218323	176
B5E11	5207580	86	229680	367
011-005	5076338	75	229700	551
001-001	5071451	101	274300	142
CES131C	5242271	163	252377	190
H219B	5079589	195	107365	435
H1447	5051027	163	100759	167
H1698	5285343	210	176589	461
196	5149407	117	264051	198
208	4823421	48	276186	239
H2262	5478956	137	197922	173
005-019	5335335	128	207935	163
H1088	5166149	119	173754	127
B1G9	5208454	92	274701	158
H2214	5136985	106	191559	176
B1A5	5245667	148	192869	143
B1H12	5171638	106	202829	157
B2B2	5253925	159	177015	177
02	5163272	184	190986	141
39	5144207	114	254637	183
183	5154965	112	201617	222
187	5198129	117	266172	153
CES103C	5083300	133	167460	151
CES9C	5116557	117	191275	152
CES164C	5045844	104	253401	124
H3084B	5030043	141	134534	169
C5	5149437	123	173754	230
TN03	5094470	79	331020	164
B5B6	5179486	98	223132	141
B12I1	5155390	100	229816	141
C23	5171332	162	201870	183
C3	5213867	167	175498	164

N50 is the sequence length of the shortest contig at 50% of the total genome length

Table S3. Primers used in this study

Assay and target region	Primer	Sequence (5'-3')	Reference
**Deletion**			
	fimB_For_FRT*kan*	AAACATAATCAGGATTAAAATGTTGGATTATTGCTAACCCAGCACAGCTAGTGCGCGTCTGTAATTATAAGGGAAAACGgtgtaggctggagctgcttc	This study
	fimB_Rev_FRT*kan*	TAAATGGTTAATCATTTTTGATATATCGTAAGAATAATGTAGTTTTTAACACCATCCCTGGTATCTCAACTATCTcatatgaatatcctccttagttcc	This study
fimB (strains S250 and CES131C)	fimB_F	AGCATGGCGTTTGTATGG	(5)
	fimB_R	CCCTGGTATCTCAACTATCTCT	(5)
	CES131CfimB_verif_F	TTTCAGGCGTTATTCAGTGC	This study
	CES131CfimB_verif_R	GATGTTGCGATTACTTCGCC	This study
	aadA_For_FRT*kan*	ATTCTGCGGTCGGCTTACCTCGCCCGTTAGACATCATGAGGGAAGCGGTGgtgtaggctggagctgcttc	This study
aadA2 (strain CES131C)	aadA_Rev_FRT*kan*	CGCGGTCGGCTTGAACGAATTGTTAGACATCATTTACCAACTGACTTGATCATAtgaatatcctccttagttc	This study
	CES131CaadA_verif_F	TTTCAGGCGTTATTCAGTGC	This study
	CES131CaadA_verif_R	GATGTTGCGATTACTTCGCC	This study
	ibeA_For_FRT*kan*	AAGCGCGGGGGATTGTTTTACTCAATTATTGAATACGGAGATAAAGTATGgtgtaggctggagctgcttc	This study
	ibeA_Rev_FRT*kan*	GGGTTTTTCTCTCATAACTTTATTCCCTGTTAAAAGACTTTTACGCCATTcatatgaatatcctccttag	This study
	ibeART_For_FTR*kan*	GCATTCTGATACAAGTTCTGAAAATGACTTGAATGGATATTATTATAATGgtgtaggctggagctgcttc	This study
ibeA et ibeART (S250)	ibeART_Rev_FTR*kan*	CGGCAAACGCATTTATTATCTTGAATAACTCAGGCCTTTGCTTCGTTGAAcatatgaatatcctccttag	This study
	S250ibeA_verif_F	TGGTGATTAGCCAGGGAGAC	This study
	S250ibeA_verif_R	AAGCGCGACATAAAAACTGG	This study
	S250ibeART_verif_F	ATTTCGCTGCACTGTTTCAC	This study
	S250ibeART_verif_R	TTTCGTGTCTTCAATGAGCG	This study
Kanamycin resistance cassette	k2	CAGTCATAGCCGAATAGCCT	(10)
	k1	CGGTGCCCTGAATGAACTGC	(10)
**Complementation**			
	fimB_cpSC-A_F	CGGACAAGATCACCCTGCGT	This study
fimB	fimBS250_cpSC-A_R	CAGGCGAATTTAAGTGATGT	This study
	fimB131C_cpSC-A_R	CAGGAGAATTTAAGTGATGT	This study
ibeA	IbeA_cpSC-A_F	GTGATGTGCTCCATAAATGCCA	This study
	ibeA_cpSC-A_R	GGCGCAACCAGTAACATACC	This study
**Sequencing**			
	Sanger_fimB_F	TCGACTTCCGGTGGTAA	This study
fimB	Sanger_fimB_R	CACACGAATGGCGTAAC	This study
	pSC-A_insert_F	AAAGGGAACAAAAGCTGGGTA	This study
	pSC-A_insert_R	GTAAAACGACGGCCAGTGAG	This study
ibeA	Sanger_ibeA_F	AGAGCACGCAACCTAAATATGG	This study
	Sanger_ibeA_R	CAGGAACCACCTAACGTCACA	This study

verif : verification

Table S4. Plasmids used in this study

Plasmid	Description	Reference
pKD4	Template plasmid for amplification of frt-flanked *kan* cassette, AmpR, KanR	(10)
pKOBEG	pBR322 derivative containing the λ Red region and *araC* gene of pKD46, CmR or TetR	(10)
pcp20	DH5α derivative containing FLP recognition, AmpR, CmR	(11)
pSC-A-amp/kan-fimB-S250	vector containing the cloned S250 wild-type *fimB* gene, AmpR, KanR	this study
pSC-A-amp/kan-fimB-CES131C	vector containing the cloned CES131C wild-type *fimB* gene, AmpR, KanR	this study
pSCA-A-amp/kan-ibeA-S250	vector containing the cloned S250 wild-type *ibeA* gene, AmpR, KanR	this study
pSC-A-amp/kan-Control_Insert	control plasmid vector, AmpR, KanR	this study

Cm: chloramphenicol,Tet: tetracycline, Amp: ampicillin, Kana: kanamycin, R: resistant

References

1. Clermont O, Couffignal C, Blanco J, Mentré F, Picard B, Denamur E, COLIVILLE and COLIBAFI groups. 2017. Two levels of specialization in bacteraemic *Escherichia coli* strains revealed by their comparison with commensal strains. Epidemiol Infect 145:872–882.

2. Leflon-Guibout V, Blanco J, Amaqdouf K, Mora A, Guize L, Nicolas-Chanoine M-H. 2008. Absence of CTX-M enzymes but high prevalence of clones, including clone ST131, among fecal *Escherichia coli* isolates from healthy subjects living in the area of Paris, France. J Clin Microbiol 46:3900–3905.

3. Lavigne J-P, Vergunst AC, Goret L, Sotto A, Combescure C, Blanco J, O'Callaghan D, Nicolas-Chanoine M-H. 2012. Virulence potential and genomic mapping of the worldwide clone *Escherichia coli* ST131. PloS One 7:e34294.

4. Pantel A, Dunyach-Remy C, Essebe CN, Mesureur J, Sotto A, Pagès J-M, Nicolas-Chanoine M-H, Lavigne J-P. 2016. Modulation of membrane influx and efflux in *Escherichia coli* sequence type 131 has an impact on bacterial motility, biofilm formation, and virulence in a *Caenorhabditis elegans* model. Antimicrob Agents Chemother 60:2901–2911.

5. Nicolas-Chanoine M-H, Petitjean M, Mora A, Mayer N, Lavigne J-P, Boulet O, Leflon-Guibout V, Blanco J, Hocquet D. 2017. The ST131 *Escherichia coli H*22 subclone from human intestinal microbiota: Comparison of genomic and phenotypic traits with those of the globally successful *H*30 subclone. BMC Microbiol 17:71.

6. Mora A, Herrera A, Mamani R, López C, Alonso MP, Blanco JE, Blanco M, Dahbi G, García-Garrote F, Pita JM, Coira A, Bernárdez MI, Blanco J. 2010. Recent Emergence of Clonal Group O25b:K1:H4-B2-ST131 ibeA Strains among Escherichia coli Poultry Isolates, Including CTX-M-9-Producing Strains, and Comparison with Clinical Human Isolates. Appl Environ Microbiol 76:6991–6997.

7. Mora A, Dahbi G, López C, Mamani R, Marzoa J, Dion S, Picard B, Blanco M, Alonso MP, Denamur E, Blanco J. 2014. Virulence patterns in a murine sepsis model of ST131 *Escherichia coli* clinical isolates belonging to serotypes O25b:H4 and O16:H5 are associated to specific virotypes. PLoS ONE 9:e87025.

8. Blanc V, Leflon-Guibout V, Blanco J, Haenni M, Madec J-Y, Rafignon G, Bruno P, Mora A, Lopez C, Dahbi G, Dunais B, Anastay M, Branger C, Moreau R, Pradier C, Nicolas-Chanoine M-H. 2014. Prevalence of day-care

centre children (France) with faecal CTX-M-producing *Escherichia coli* comprising O25b:H4 and O16:H5 ST131 strains. J Antimicrob Chemother 69:1231–1237.

9. Vimont S, Boyd A, Bleibtreu A, Bens M, Goujon J-M, Garry L, Clermont O, Denamur E, Arlet G, Vandewalle A. 2012. The CTX-M-15-producing *Escherichia coli* clone O25b:H4-ST131 has high intestine colonization and urinary tract infection abilities. PloS One 7:e46547.

10. Datsenko KA, Wanner BL. 2000. One-step inactivation of chromosomal genes in *Escherichia coli* K-12 using PCR products. Proc Natl Acad Sci U S A 97:6640–6645.

11. Cherepanov PP, Wackernagel W. 1995. Gene disruption in *Escherichia coli*: TcR and KmR cassettes with the option of Flp-catalyzed excision of the antibiotic-resistance determinant. Gene 158:9–14.
