# Supplementary material for: Success of Escherichia coli O25b:H4 Sequence Type 131 Clade C Associated with a Decrease in Virulence
Source: Infect Immun. 2020 Nov 16;88(12):e00576-20. doi: 10.1128/IAI.00576-20 (PMC7671891; doi:10.1128/IAI.00576-20)
Supplement: Supplemental file 2 [file IAI.00576-20-s0002.pdf]

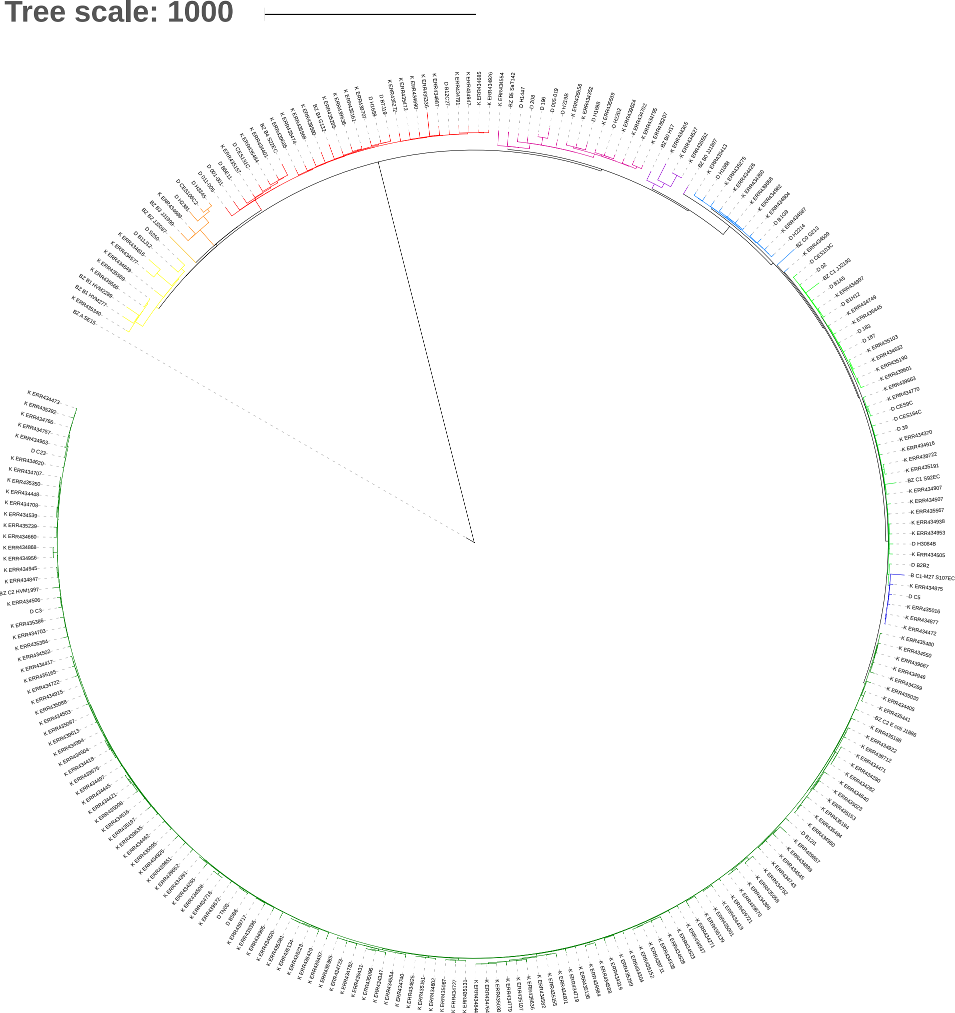
Tree scale: 1000
K ERR434473
K ERR435392
K ERR434766
K ERR434757
K ERR434963
D C23
K ERR434620
K ERR434707
K ERR435350
K ERR434448
K ERR434708
K ERR434539
K ERR435239
K ERR434660
K ERR434868
K ERR434956
K ERR434945
K ERR434847
BZ C2 HVM1997
K ERR434506
D C3
K ERR435386
K ERR434703
K ERR435384
K ERR434502
K ERR434417
K ERR435165
K ERR434722
K ERR434915
K ERR435088
K ERR434503
K ERR435087
K ERR439613
K ERR434994
K ERR434504
K ERR434418
K ERR439575
K ERR434497
K ERR434445
K ERR434421
K ERR435008
K ERR434516
K ERR435197
K ERR439635
K ERR434462
K ERR435095
K ERR434925
K ERR439651
K ERR439652
K ERR434391
K ERR434265
K ERR434508
K ERR434716
K ERR439672
D TN03
D B5B6
K ERR439717
K ERR435395
K ERR434995
K ERR434520
K ERR435081
K ERR435134
K ERR435228
K ERR435429
K ERR435457
K ERR435385
K ERR434723
K ERR434782
K ERR435431
K ERR435096
K ERR434347
K ERR434584
K ERR434740
K ERR434825
K ERR435151
K ERR434602
K ERR435067
K ERR434727
K ERR435131
K ERR434844
K ERR434764
K ERR435030
K ERR434779
K ERR435107
K ERR439636
K ERR434582
K ERR435155
K ERR434601
K ERR434719
K ERR435138
K ERR439564
K ERR434588
K ERR434319
K ERR435289
K ERR434304
K ERR435152
K ERR439711
K ERR434338
K ERR434528
K ERR434523
K ERR434937
K ERR434271
K ERR435139
K ERR435001
K ERR434419
K ERR439721
K ERR439670
K ERR434368
K ERR435058
K ERR434752
K ERR434743
K ERR434545
K ERR434898
K ERR439657
D B121
K ERR434960
K ERR435494
K ERR435194
K ERR435153
K ERR435023
K ERR434640
K ERR434282
K ERR434280
K ERR434471
K ERR439712
K ERR434922
K ERR435188
BZ C2 E coli J1886
K ERR435441
K ERR434405
K ERR435020
K ERR434269
K ERR434946
K ERR439667
K ERR434550
K ERR435480
K ERR434472
K ERR434877
K ERR435016
D C5
K ERR434875
B C1-M27 S107EC
D B2B2
K ERR434505
D H3084B
K ERR434953
K ERR434938
K ERR435567
K ERR434507
K ERR434907
BZ C1 S92EC
K ERR435191
K ERR439722
K ERR434916
K ERR434370
D 39
D CES164C
D CES9C
K ERR434770
K ERR439663
K ERR439601
K ERR435190
K ERR434632
K ERR435103
D 187
D 183
K ERR435445
K ERR434749
D B1H12
K ERR434997
D B1A5
BZ C1 JJ2193
D 02
D CES103C
K ERR434509
BZ C0 G213
D H2214
K ERR434587
D B1G9
K ERR434804
K ERR434982
K ERR439658
K ERR434350
K ERR434426
K ERR435275
D H1088
K ERR435413
BZ B0 JJ1897
K ERR435552
K ERR434527
K ERR434365
BZ B0 H17
K ERR435207
K ERR434795
K ERR434702
K ERR439624
D H2262
K ERR435539
D H1698
K ERR434352
K ERR435556
D H219B
D 005-019
D 196
D 208
D H1447
BZ B5 SaT142
K ERR434554
K ERR434926
K ERR434685
K ERR434947
K ERR434791
D B12C27
K ERR434987
K ERR435336
K ERR434690
K ERR435472
K ERR435272
D B7J19
D H1659
K ERR439707
K ERR435161
K ERR439638
K ERR435285
BZ B4 G132
K ERR439590
K ERR435568
K ERR435474
K ERR439685
BZ B4 S22EC
K ERR434401
K ERR435484
D CES131C
K ERR435157
D B5E11
D 001-001
D 011-005
D H3345
D CES106C2
D H2381
K ERR434699
BZ B3 JJ1999
BZ B2 JJ2087
D S250
D B11J12
K ERR434616
K ERR434577
K ERR434649
K ERR435569
K ERR435566
BZ B1 HVM2289
BZ B1 HVM277
K ERR435340
BZ A SE15

Figure S1. Maximum likelihood phylogenetic tree. This tree was built from non-recombinant SNPs using maximum likelihood, rooted on a clade A ST131 reference strain, SE15. Scale bar represents the average nucleotide substitutions per site. Branches are colored in accordance with subclades: B1 in yellow, B2 in pale orange, B3 in dark orange, B4 in red, B5 in pink, B0 in purple, C0 in pale blue, C1 in light green, C1-M27 in dark blue and C2 in dark green. Strains are named according to their origin: BZ for Ben Zakour et al. (1), K for Kallonen et al. (2) and D for this work.

References

1. Ben Zakour NL, Alsheikh-Hussain AS, Ashcroft MM, Khanh Nhu NT, Roberts LW, Stanton-Cook M, Schembri MA, Beatson SA. 2016. Sequential acquisition of virulence and fluoroquinolone resistance has shaped the evolution of *Escherichia coli* ST131. MBio 7:e00347-00316.

2. Kallonen T, Brodrick HJ, Harris SR, Corander J, Brown NM, Martin V, Peacock SJ, Parkhill J. 2017. Systematic longitudinal survey of invasive *Escherichia coli* in England demonstrates a stable population structure only transiently disturbed by the emergence of ST131. Genome Res 27:1437–1449.
