# Supplementary material for: Success of Escherichia coli O25b:H4 Sequence Type 131 Clade C Associated with a Decrease in Virulence
Source: Infect Immun. 2020 Nov 16;88(12):e00576-20. doi: 10.1128/IAI.00576-20 (PMC7671891; doi:10.1128/IAI.00576-20)
Supplement: Supplemental file 3 [file IAI.00576-20-s0003.pdf]

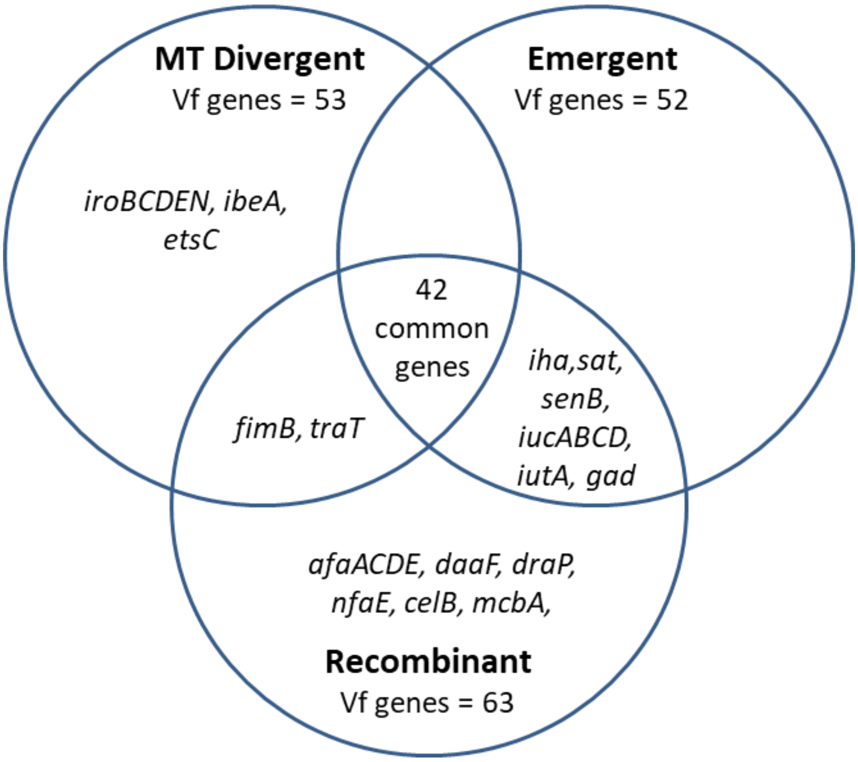

Figure S2. Virulence factors (VF)-encoding genes in MT Divergent, Emergent and Recombinant. Each strain is represented by a circle. VF-encoding genes specific of a strain are indicated in the unshared part of the circle, while genes in common are indicated in the intersecting region between the two or three involved strains. Among the 67 genes found at least once in these three strains, the 42 common genes are as follows: *fdeC, fimACDEFGHI, yfcV, yagVWXYZ, ykgK, chuA, fyuA, entABCEFS, fepABCDG, fes, irp2, kpsDEMMTII, K5, iss, malX, usp, ompT, ompA and aslA.*
