# Supplementary material for: Success of Escherichia coli O25b:H4 Sequence Type 131 Clade C Associated with a Decrease in Virulence
Source: Infect Immun. 2020 Nov 16;88(12):e00576-20. doi: 10.1128/IAI.00576-20 (PMC7671891; doi:10.1128/IAI.00576-20)
Supplement: Supplemental file 4 [file IAI.00576-20-s0004.pdf]

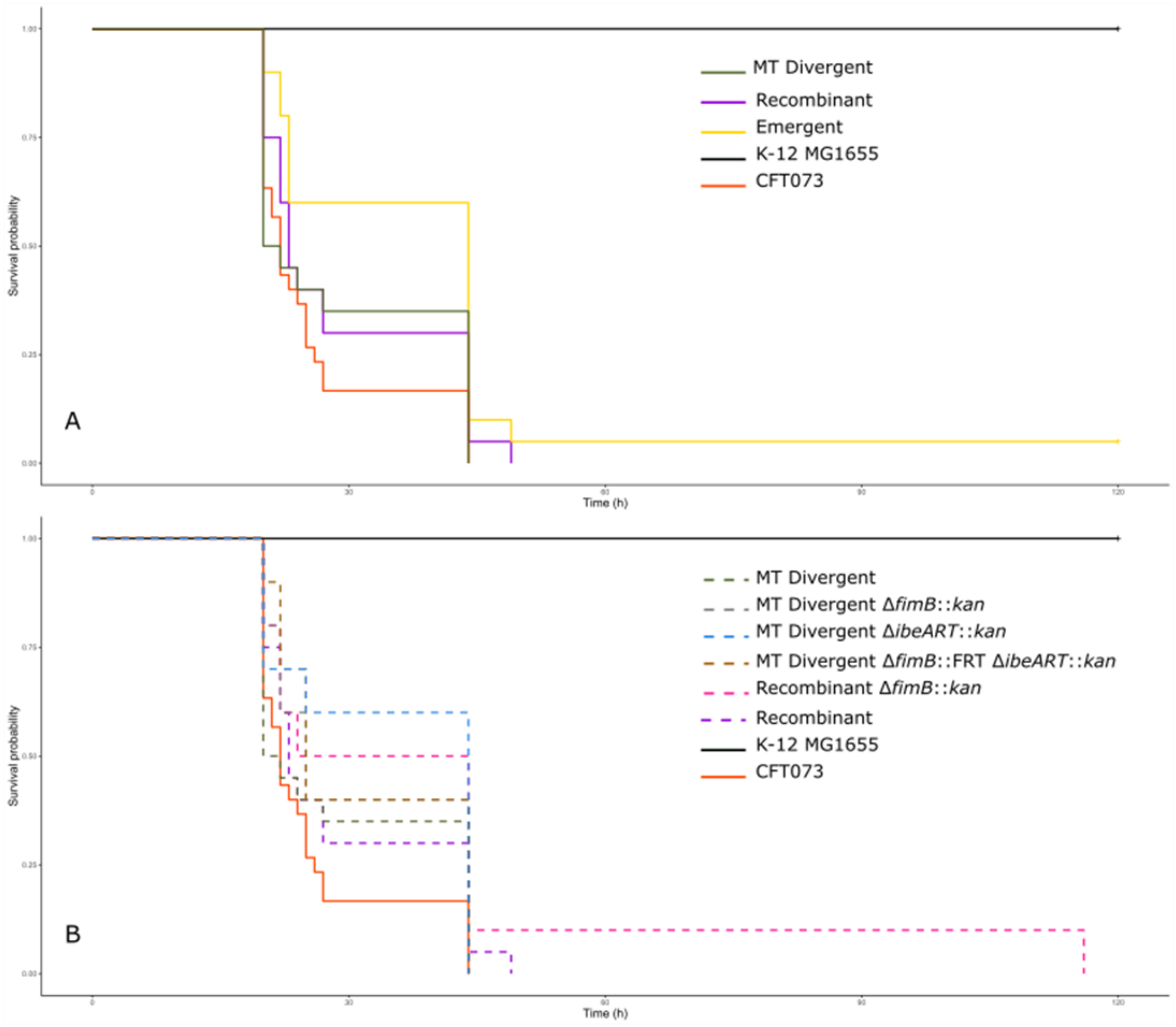

Figure S3. Kaplan-Meier survival curves of MT Divergent, Recombinant, Emergent and mutants of MT Divergent and Recombinant in mono infection assay in the mouse sepsis model

The tested strains and controls (positive: CFT073, negative: K12-MG1655) are represented by different colored lines. **A**. Kaplan Meier survival curves of MT Divergent, Recombinant, Emergent. MT Divergent (20 mice) killed mice significantly faster than Emergent (20 mice) (P=0.04). but not than Recombinant (20 mice) (P=0.8). Recombinant and Emergent killed mice similarly (P=0.06). **B**. Kaplan Meier survival curves of mutants. There was no significant difference neither between MT Divergent (20 mice) and its Δ*fimB*::*kan* (10 mice), Δ*ibeART*:: *kan* (10 mice) and *ΔfimB*::FRT Δ*ibeART*::*kan* (10 mice) mutants nor between Recombinant (20 mice) and its Δ*fimB*::*kan* mutant (10 mice).
