# Supplementary material for: Success of Escherichia coli O25b:H4 Sequence Type 131 Clade C Associated with a Decrease in Virulence
Source: Infect Immun. 2020 Nov 16;88(12):e00576-20. doi: 10.1128/IAI.00576-20 (PMC7671891; doi:10.1128/IAI.00576-20)
Supplement: Supplemental file 5 [file IAI.00576-20-s0005.pdf]

Success of *Escherichia coli* O25b:H4 ST131 clade C associated with a decrease in virulence

Marion DUPRILOT[a,b‡], Alexandra BARON[a‡], François BLANQUART[a,c], Sara DION[a], Cassandra POUGET[d], Philippe LETTÉRON[e], Saskia-Camille FLAMENT-SIMON[f], Olivier CLERMONT[a], Erick DENAMUR[a,g], Marie-Hélène NICOLAS-CHANOINE[a]#

[a] Université de Paris, INSERM, IAME, 75018 Paris, France

[b] AP-HP, Laboratoire de Microbiologie, Hôpital Beaujon, 92110 Clichy, France

[c] Centre for Interdisciplinary Research in Biology (CIRB), Collège de France, CNRS, INSERM, PSL Research University, 75005 Paris, France

[d] VBMI, INSERM U1047, Université de Montpellier, Nîmes, France

[e] Université de Paris UMR 1149 INSERM-ERL CNRS 8252, 75018 Paris, France

[f] Laboratorio de Referencia de *Escherichia coli* (LREC), Departamento de Microbioloxía e Parasitoloxía, Facultade de Veterinaria, Universidade de Santiago de Compostela (USC), Lugo, Spain.

[g] AP-HP, Laboratoire de Génétique Moléculaire, Hôpital Bichat, 75018 Paris, France

‡MD and AB share first authorship on this work

#Address correspondence to:

Marie-Hélène NICOLAS-CHANOINE, e-mail: marie-helene.nicolas-chanoine@inserm.fr

Technical details on Materials and Methods

Bacterial strains

Thirty-nine O25:H4 ST131 *Escherichia coli* isolates were specifically collected for the present study. The 18 *fimH*22 strains had been isolated between 1993 and 2011 in Spain (n=7) and Ile de France (n=11) from the blood of adult patients (n=11) and the feces of heathy

adults (n=7). The 21 *fimH*30 strains had been isolated during the same period in Spain (n=3) and in Ile de France (n=18) from the blood (n=9) and the urinary tract (n=1) of adults patients and the feces of health adults (n=8) and children (n=3). Four of the 18 *fimH*22 strains were resistant to nalidixic acid whereas 17 of the 21 *fimH*30 strains were resistant to both nalidixic acid and ciprofloxacin according to the agar disk diffusion method interpreted following the EUCASTrecommendations (www.eucast.org). Three of the 18 *fimH*22 strains and six of the 21 *fimH*30 strains were extended-spectrum β-lactamase (ESBL) producers according to the double disk synergy test (1). The 39 strains were stored in 20% glycerol at -80°C until use. *E. coli* CFT073 and *E. coli* K-12 MG1655 strains were used as positive and negative controls, respectively, in the sepsis mouse model, and *E. coli* UTI89 and *E. coli* UBA83972 strains as positive and negative controls, respectively, in the yeast agglutination assays.

Genome sequencing and analysis

Whole genome sequencing (WGS) of the 39 strains was performed. Total DNA was extracted using NucleoMag Tissue (Macherey-Nagel, Düren, Germany). Libraries were prepared using Nextera technology and sequenced with an Illumina HiSeq (IntegraGen Genomics, Evry, France) using a 2x100 base pair (bp) paired-end strategy. Reads were assembled with SPAdes (2). We investigated how the different subclades B and C of ST131 was represented in our collection and how they evolved in frequency over time in a larger collection from established by Kallonen *et al.* (3). To that end, we complemented the studied 39 genomes with 218+21 genomes from two published studies [218 from Kallonen *et al.* (3), 21 from Ben Zakour *et al.* (4)]. We annotated the genomes using Prokka (5), identified the core genome using Roary (6), and inferred the phylogenetic history of all these strains after removing the identified recombination using Gubbins (7) and the phylogenetic software RAxML (8). Outlier sequences were removed. The tree was then viewed and modified using iTOL (9). From this

phylogeny, we identified sequences belonging to different subclades B0-B5 and C0-C2 of O25b:H4 ST131, as defined in Ben Zakour *et al.* (4).

Gene deletion and complementation

Primers and plasmids used for gene deletion and complementation are listed in Table S3 and S4, respectively. Replacement by a kanamycin resistance cassette was used to inactivate the chromosomal *fimB* gene of S250 (called MT Divergent) and CES131C (called Recombinant) strains, the chromosomal *ibeA* gene and *ibeART* operon of MT Divergent, and the plasmid-mediated *aadA2* gene encoding streptomycin resistance in Recombinant following a strategy adapted from Datsenko and Wanner (10). Briefly, the kanamycin resistance cassette was amplified from pKD4 using primers containing 50-79 nucleotide flanking regions complementary to the beginning and end of the *fimB*, *ibeA* and *aadA2* genes and of the *ibeART* operon. The knock-out (KO) PCR product was introduced by electroporation into competent cells transformed with a tetracycline or chloramphenicol resistant pKOBEG plasmid carrying the λ-Red recombinase. When necessary, the kanamycin-encoding gene was eliminated from Δ*fimB*::*kan*, Δ*ibeA*::*kan* and Δ*ibeART*::*kan* mutants resulting in Δ*fimB*::FRT Δ*ibeA*::FRT and Δ*ibeART*::FRT mutants as previously described (10). Complementation of Δ*fimB*::FRT and Δ*ibe*A::FRT mutants were performed by cloning the promoter and encoding regions of the parental *fimB* and *ibeA* genes into pSC-A-amp/kan by using the StrataClone PCR Cloning Kit (Agilent Technologies, Massy, France). The recombinant plasmid was then electroporated into competent MT Divergent Δ*fimB*::FRT, MT Divergent Δ*ibeA*::FRT and Recombinant Δ*fimB*::FRT strains, and transformants were selected on lysogenic Broth (LB) (Invitrogen, Carlsbad, California, USA) agar plates containing 100 mg/L of kanamycin. The empty plasmid pSC-A-amp/kan-Control_Insert was used as negative control. All mutants were confirmed by PCR and sequencing. Because of the absence of genes encoding antibiotic resistance in MT Divergent, we used the *kan* gene of the kanamycin resistance cassette when

MT Divergent was put in competition with its Δ*fimB*::*kan*, Δ*ibeA*::*kan* and Δ*ibeART*::*kan* mutants in order to be able to assess the number of CFU/ml for each strain. In the double MT Divergent Δ*fimB*::FRT Δ*ibeART*::*kan* mutant, the kanamycin cassette was removed from the Δ*fimB* gene but conserved in the Δ*ibeART* operon. The same process was used for Recombinant when it was put in competition with its Δ*fimB*::*kan* mutant.

Kinetics of early biofilm formation

The primary step of biofilm formation was measured in the 39 strains by using BioFilm Ring Test® (BioFilm Control, Saint-Beauzire, France) according to the manufacturer's recommendations and as previously described (11). Briefly, each strain was sub-cultured twice on brain heart infusion (BHI) agar (Benton Dickinson, Le Pont-de-Claix, France) at 37°C for 24 h. Three colonies of the second subculture were suspended in BHI broth (Biofilm Control) and density at 600 nm (OD600) was measured (Ultrospec10: Biochrom, Cambridge, UK). An adequate volume of this suspension was then added to a mixture (1% vol/vol) of BHI broth and magnetic microbeads (TONER 4, Biofilm Control) to obtain a final concentration of approximately 10^6 CFU/ml. 200 µl of this mix were deposited twice in three polystyrene 96-well microtiter plates that were incubated during 2, 3 and 5 h, respectively. At the end of each incubation time, 100 µl of liquid contrast solution (LIC001, Biofilm Control) was added on the top of each well and the microplate was put on a magnetic block for 1 min. This magnet support is made up of 96 minimagnets centered under the bottom of each well. During magnet contact, free beads were attracted toward the center of the wells, forming a brown spot, while beads embedded in biofilm were blocked and remained undetectable. The microplate was then scanned using a BioFilm Control plate reader. The intensity of the spot was analyzed using the BioFilm Ring Test® software version 3.0.3 and expressed as the biofilm formation index (BFI), with values ranging from 20 (absence of biofilm formation) to 0 (high biofilm formation), inversely proportional to the biofilm formation ability. The

experiments were repeated at least three times for each strain. BHI broth was used as negative control, and strains S250 and 39, previously described with this method as early and never biofilm producers, respectively, as control strains (12).

Expression of type 1 fimbriae

Expression of type 1 fimbriae was assessed in the 39 strains and mutants by using the yeast cell (*Saccharomyces cerevisiae*) agglutination assay as previously described (13), Briefly, for each strain, five single colonies were inoculated into 3 ml of LB and incubated for 24 h in shaking and static conditions. Agglutination was performed by mixing 50 µL of yeast cells (diluted to 5% in phosphate buffer saline (PBS) and 50 µL of bacterial culture on a glass slide. To study the early expression of type 1 fimbriae, the same protocol was applied to cultures incubated for 2 and 5 h in shaking conditions. To this end, 50 µL of yeast cells were mixed with the 50 µL-pellet (obtained after centrifugation: 3000g for10 min at 4°) of the 2 h-shaking LB culture and 50 µL of the 5 h-shaking LB culture. Bacterial-yeast aggregation was macroscopically observed and a bacterial strain was considered negative for type 1 fimbriae expression when no visible aggregation was produced after 3 min.

Maximum growth rate

Fitness assay was performed for the 39 strains as previously described in LB (14). Strains were grown overnight (O/N) in LB at 37°C under continuous stirring at 200 rpm. O/N cultures were washed three times in a physiological saline solution, and, after optical density (OD) adjustment, were inoculated twice at 1/10000 in a Costar® 96 flat-bottomed well plate. The plate was incubated in an automatic spectrophotometer (Tecan Infinite F200 Pro) that measures the OD_{600} in each well every 5 min over a period of 24 h. The experiment was repeated three times. Growth curves were then analyzed and maximum growth rates was calculated and expressed in h^{-1}. We decided to explore only the maximum growth rate, because contrary to the lag time (*i.e.* time required to reach the exponential growth phase) and

the final optical density, the maximum growth rate does not depend on the initial number of bacteria and on the well position, and is less affected by the form of the growth curve (15).

Mouse models

Mono infection assay and competition assay in the sepsis model

Female mice OF1 of 14-16 g (4-week-old) from Charles River® were used to assess the individual strain ability and, in competition assays, the strains' relative ability to cause sepsis, as previously described (16). Briefly, 200 µl of a suspension of 10^9 *E. coli*/mL in physiological serum was inoculated by subcutaneous injection in the neck, either alone or mixed at a ratio of 1:1. Time to death was monitored during the following seven days, and all mice were frozen at -20°C at time of death. Mice surviving after 7 days were considered cured and were sacrificed. From ten to twenty mice were used for the strains tested alone and from five to ten for the strains tested in competitions. CFT073 strain, known to quickly kill any inoculated mouse, was used as positive control, and *E. coli* K-12 MG1655 strain, unable to kill any inoculated mouse as negative control (17) in the individual strain assays. Kaplan-Meier curves of mouse survival were performed and, in competition assays, the spleen of all spontaneously dead mice was collected, weighed, pounded in physiological water and dilutions of spleen suspensions were plated on LB agar and LB agar with appropriate antibiotics (kanamycin 50 mg/L, ciprofloxacin 1 mg/L or ampicillin 100 mg/L) to estimate the infecting populations. Competitive indexes (CI) were obtained using the following formula: logCFU[(isolate1/isolate2)T_x/(isolate1/isolate2)T_0], isolate1 being the first strain cited and isolate2 the second one in the figure legends.

Competition assay in the intestinal colonization model

Six-week-old female mice CD-1 from Charles River® (L'Arbresle, France) pre-treated with streptomycin before inoculation of challenging strains were used to assess strains' relative ability to colonize the mouse intestine, as previously described (14). Briefly, mice received water

supplemented with streptomycin sulfate (5 g/liter) for five days in order to eliminate *E. coli* from gut before studied strain inoculation, and were fed from then with sterile food until the end of the experiment. Streptomycin treatment was stopped five days before studied strain inoculation to allow the subsequent colonization of the mouse intestine by streptomycin-sensitive strains. At inoculation day, about 10^6 *E. coli* mixed at a 1:1 ratio were administrated by force-feeding in 200 µL of physiological water to mice free of coliform flora (controlled by plating the feces on Drigalski plates). At days 1, 4 and 7, the intestinal population of *E. coli* was estimated by plating dilutions of weighed fresh feces on LB agar and LB agar with appropriate antibiotics (kanamycin 50 mg/L or ciprofloxacin 1 mg/L). At least five mice were used for each competition assay. CIs were obtained as described above

<u>Competition assay in the urinary tract infection model</u>

CBA female mice of 8-22g (8-week-old) from Janvier® (Le Genest-Saint-Isle, France) were used to assess strains' relative ability to cause an ascending unobstructed urinary tract infection, as previously described (18). Briefly, following a 12 h hydric restriction, anesthetized mice (using a xylazine/ketamine mixture) were infected with about 10^8 *E. coli* mixed at a 1:1 ratio in 50 µL of physiological water, *via* the transurethral route into the bladder. Once the anesthetic effect achieved, hydric restriction was stopped. Mice were sacrificed 48 h after the bacterial inoculation. The bladder and the kidneys were aseptically collected, weighed and pounded in 1 mL of physiological water. The populations of *E. coli* were estimated by plating dilutions of the bladder and kidneys suspensions on LB agar and/or LB agar with appropriate antibiotics (ciprofloxacin 1 mg/L or ampicillin 100 mg/L). Ten mice were used for each competition assay. Mice were considered infected if at least one colony grew on the kidneys' LB agar plates, which corresponds to approximately 60-70 CFU/g of the kidneys. CIs were obtained as described above. In the absence of colony on LB agar with antibiotics, CI was

calculated by considering the growth of five colonies, which is under the method detection limit and corresponds to about 30 CFU/g of the kidneys.

References

1. Jarlier V, Nicolas MH, Fournier G, Philippon A. 1988. Extended broad-spectrum beta-lactamases conferring transferable resistance to newer beta-lactam agents in Enterobacteriaceae: hospital prevalence and susceptibility patterns. Rev Infect Dis 10:867–878.

2. Bankevich A, Nurk S, Antipov D, Gurevich AA, Dvorkin M, Kulikov AS, Lesin VM, Nikolenko SI, Pham S, Prjibelski AD, Pyshkin AV, Sirotkin AV, Vyahhi N, Tesler G, Alekseyev MA, Pevzner PA. 2012. SPAdes: a new genome assembly algorithm and its applications to single-cell sequencing. J Comput Biol J Comput Mol Cell Biol 19:455–477.

3. Kallonen T, Brodrick HJ, Harris SR, Corander J, Brown NM, Martin V, Peacock SJ, Parkhill J. 2017. Systematic longitudinal survey of invasive *Escherichia coli* in England demonstrates a stable population structure only transiently disturbed by the emergence of ST131. Genome Res 27:1437–1449.

4. Ben Zakour NL, Alsheikh-Hussain AS, Ashcroft MM, Khanh Nhu NT, Roberts LW, Stanton-Cook M, Schembri MA, Beatson SA. 2016. Sequential acquisition of virulence and fluoroquinolone resistance has shaped the evolution of *Escherichia coli* ST131. mBio 7:e00347-00316.

5. Seemann T. 2014. Prokka: rapid prokaryotic genome annotation. Bioinforma Oxf Engl 30:2068–2069.

6. Page AJ, Cummins CA, Hunt M, Wong VK, Reuter S, Holden MTG, Fookes M, Falush D, Keane JA, Parkhill J. 2015. Roary: rapid large-scale prokaryote pan genome analysis. Bioinforma Oxf Engl 31:3691–3693.

7. Croucher NJ, Page AJ, Connor TR, Delaney AJ, Keane JA, Bentley SD, Parkhill J, Harris SR. 2015. Rapid phylogenetic analysis of large samples of recombinant bacterial whole genome sequences using Gubbins. Nucleic Acids Res 43:e15–e15.

8. Stamatakis A. 2006. RAxML-VI-HPC: maximum likelihood-based phylogenetic analyses with thousands of taxa and mixed models. Bioinformatics 22:2688–2690.

9. Letunic I, Bork P. 2019. Interactive Tree Of Life (iTOL) v4: recent updates and new developments. Nucleic Acids Res 47:W256–W259.

10. Datsenko KA, Wanner BL. 2000. One-step inactivation of chromosomal genes in *Escherichia coli* K-12 using PCR products. Proc Natl Acad Sci U S A 97:6640–6645.

11. Flament-Simon S-C, Duprilot M, Mayer N, García V, Alonso MP, Blanco J, Nicolas-Chanoine M-H. 2019. Association between kinetics of early biofilm formation and clonal lineage in *Escherichia coli*. Front Microbiol 10:1183.

12. Nicolas-Chanoine M-H, Petitjean M, Mora A, Mayer N, Lavigne J-P, Boulet O, Leflon-Guibout V, Blanco J, Hocquet D. 2017. The ST131 *Escherichia coli H*22 subclone from human intestinal microbiota: Comparison of genomic and phenotypic traits with those of the globally successful *H*30 subclone. BMC Microbiol 17:71.

13. Totsika M, Beatson SA, Sarkar S, Phan M-D, Petty NK, Bachmann N, Szubert M, Sidjabat HE, Paterson DL, Upton M, Schembri MA. 2011. Insights into a multidrug

resistant *Escherichia coli* pathogen of the globally disseminated ST131 lineage: genome analysis and virulence mechanisms. PLOS ONE 6:e26578.

14. Vimont S, Boyd A, Bleibtreu A, Bens M, Goujon J-M, Garry L, Clermont O, Denamur E, Arlet G, Vandewalle A. 2012. The CTX-M-15-producing *Escherichia coli* clone O25b:H4-ST131 has high intestine colonization and urinary tract infection abilities. PloS One 7:e46547.

15. Bleibtreu A, Gros P-A, Laouénan C, Clermont O, Le Nagard H, Picard B, Tenaillon O, Denamur E. 2013. Fitness, stress resistance, and extraintestinal virulence in *Escherichia coli*. Infect Immun 81:2733–2742.

16. Smati M, Magistro G, Adiba S, Wieser A, Picard B, Schubert S, Denamur E. 2017. Strain-specific impact of the high-pathogenicity island on virulence in extra-intestinal pathogenic *Escherichia coli*. Int J Med Microbiol IJMM 307:44–56.

17. Johnson JR, Clermont O, Menard M, Kuskowski MA, Picard B, Denamur E. 2006. Experimental mouse lethality of *Escherichia coli* isolates, in relation to accessory traits, phylogenetic group, and ecological source. J Infect Dis 194:1141–1150.

18. Labat F, Pradillon O, Garry L, Peuchmaur M, Fantin B, Denamur E. 2005. Mutator phenotype confers advantage in *Escherichia coli* chronic urinary tract infection pathogenesis. FEMS Immunol Med Microbiol 44:317–321.
